# Query-guided learning for efficient and interpretable multi-class brain tumor classification in MRI

**DOI:** 10.3389/frai.2026.1849051

**Published:** 2026-06-22

**Authors:** B. Uthra, R. Sivashankari

**Affiliations:** School of Computer Science Engineering and Information Systems, Vellore Institute of Technology, Vellore, Tamil Nadu, India

**Keywords:** brain tumor classification, deep learning, explainable AI, learnable query tokens, lightGBM, medical imaging, Query-Guided Cross-Attention CNN, stacked ensemble

## Abstract

**Introduction:**

Accurate and efficient multi-class classification for brain tumors from MRI images is still a significant problem, requiring not only local feature learning but also global context comprehension.

**Methods:**

We present a novel Query-Guided Cross-Attention Convolutional Neural Network (QGC-CNN), which incorporates convolutional learning into a transformer-style network with learnable query tokens. The interaction between query tokens and feature maps helps discover long-range relationships that are difficult for standard CNNs to capture. To improve reliability and robustness, an ensemble method combining Xception, EfficientNetB0, ResNet50, and QGC-CNN was constructed, where the predicted values were combined using a LightGBM meta-learner.

**Results:**

The experiments show that the proposed framework achieved a test accuracy of 95.50% and an AUC of 0.99 for the “No Tumor” class. Importantly, the QGC-CNN model shows great efficiency, having just 0.83 GFLOPs, which is 10.9 times lower than that of Xception.

**Discussion:**

The proposed framework demonstrates that combining query-guided cross-attention with ensemble learning can provide accurate and computationally efficient brain tumor classification from MRI images.

## 1 Introduction

Brain tumors remain one of the most critical challenges in medical imaging, necessitating accurate and early detection for effective treatment. The classic way of manual diagnosis from MRI is time-consuming, subject to inter-observer variability and sometimes error-prone. This motivates the need for automated computer-aided diagnostic systems. Deep learning (DL), a subset of Machine Learning, particularly convolutional neural networks (CNNs), has shown excellent performance in medical imaging tasks, yet CNNs often lack the capacity to capture long-range dependencies. Vision transformers (ViTs) offer global feature modeling but require large datasets and are computationally expensive. Furthermore, the integration of transformer-based architectures with CNNs (Chen et al., 2021; Liu et al., 2021) offers a promising avenue for capturing local as well as global features in medical images. This work is motivated by the hypothesis that an ensemble of diverse and powerful models can lead to a more robust and accurate classification system. This study proposes a Query-Guided Cross-Attention CNN (QGC-CNN) framework that combines the local feature extraction power of CNNs with transformer-based global attention using learnable query tokens. Furthermore, the robustness is enhanced by employing an ensemble of diverse CNN backbones such as Xception (Chollet, 2017), EfficientNetB0 (Tan and Le, 2019), and ResNet50 (He et al., 2016) and the custom QGC-CNN. For

Then, the predictions are fused through a LightGBM meta-classifier (Ke et al., 2017) trained on cross-validation outputs, yielding improved generalization.

Despite remarkable progress, current solutions suffer from three major drawbacks:

1. The CNN-based approach is limited due to the receptive field and is not capable of modeling long-range dependencies;
2. The transformer-based solution comes at a computational cost;
3. the ensemble approach has no reliable calibration mechanism.

In order to overcome these obstacles, few contributions were performed and are summarized as follows:

1. This work introduces a new hybrid model that utilizes the combination of convolutions to extract features along with transformer-like attention mechanisms through the use of learnable query tokens. The query tokens interact with the feature maps to identify long-range spatial correlations for a discriminative representation of the tumors rather than the classical attention approach.
2. The model successfully exploits the power of CNNs for extracting local textural information and attention for global reasoning and demonstrates a competitive performance balance between efficiency and performance (0.83 GFLOPs).
3. A heterogeneous ensemble model consisting of Xception, ResNet50, EfficientNetB0, and QGC-CNN is fused via the LightGBM meta-learner.
4. Effectiveness of this architecture is analyzed using classification accuracy, computation cost (FLOPs), and probability calibration. Additionally, the interpretation of Grad-CAM (Selvaraju et al., 2017) and attention queries helps to understand how the architecture works internally. The proposed model demonstrates very encouraging results for classification of different brain tumors from multi-class MRI dataset.

The rest of the paper is organized as follows. Section 2 provides a review of the relevant work, Section 3 proposes the methodology, Section 4 shows the experimental results, and Section 5 demonstrates the findings and implications of this study and Section 6 draws conclusions to the paper.

## 2 Related works

The diagnosis of brain tumors with MRI has been extensively investigated in existing research. Recent advances in DL have greatly improved brain tumor classification and segmentation. Existing research can be classified into CNN-based classification, attention-based models, and hybrid models. Most of the existing research has used CNN-based classification. In (Nassar et al. 2024), 3,064 T1-weighted contrast-enhanced MRI images of 233 subjects were used to classify brain tumors into meningioma, glioma, and pituitary tumor types. A 3D CNN-based classification was performed. In another existing research (Hosny et al., 2025), an ensemble of five CNN models with voting strategy is proposed. They used 3,064 MRI images of 233 subjects and achieved an accuracy of 99.31%. Models such as DenseNet121 and InceptionV3 models with transfer learning were used.

In order to solve the problem of complexity in multi-class classification, (Golkarieh et al. 2025) used large-scale classification with 17 classes of tumor data. The models such as VGG19, ResNet50, InceptionV3, and EfficientNetV2, and an adaptive fine-tuning strategy in two stages were used. In this case, EfficientNetV2 performed better in terms of accuracy, with an accuracy of 98.22%, and precision, recall, and F1-score metrics.

In addition, other researchers have used attention mechanisms in order to solve some of the complexities in image classification. For example, (Muksimova et al. 2025) proposed an attention mechanism in which a lightweight YOLOv5m model with an Enhanced Spatial Attention (ESA) module is used. In this case, the proposed model performed better in image detection compared to other models in detecting objects in the Figshare dataset. Similarly, technique named “Variational Spatial Attention with Graph Convolutional Neural Network” (Joshi et al., 2025) combining variational spatial attention, graph convolutional networks, and recurrent units was developed. This obtained high accuracy on the BraTS 2019 dataset while reducing computational time.

At the same time, segmentation-based methods have also been extensively explored. The study in (Hussain et al. 2025) compared and evaluated UNet, Attention-UNet, and Residual-Attention-UNet models. Out of all, the Residual-Attention-UNet model showed better performance in terms of segmentation (Dice Coefficient: 91.10%) and classification. Moreover, transfer learning-based CNN model namely modified VGG-19 models, has also been proposed in (Nancy and Maheswari 2025); (Prabhas et al. 2025), thus highlighting the ability of pre-trained models for efficient medical imaging applications. Although these methods achieve high accuracy, they often suffer from limitations such as high computational cost, limited global context modeling, and unreliable probability calibration. Despite these developments, recent studies have emphasized the improvement of the performance of multi-class classification while maintaining computational efficiency. A comparative summary of these state-of-the-art approaches is provided in Table 1. The datasets, algorithms are the key aspects considered in the comparison. The Table 2 gives an analysis of performance metrics, and research gaps identified from the literature.

**Table 1:** Comparison of recent brain tumor classification methods highlighting datasets, model types, and key algorithmic approaches used in prior studies.

References	Dataset	Algorithm
Lai et al. (2025)	4,449 MRI slices (6 classes)	Vision Mamba (Vim); compared with ViT, Swin, EfficientNet-B0
Maloji et al. (2024)	Figshare; 3,064 T1 MRI scans (3 classes)	Optimized ResNet50 with identification blocks
Zarenia et al. (2025)	Kaggle dataset (15 classes)	Multiscale Deformable Attention Module (MS-DAM) with DCN
Heythem et al. (2024)	T1, T2, FLAIR MRI datasets	Custom CNN; compared with VGG16, VGG19, ResNet
Tariq et al. (2025)	Combined Kaggle datasets; 7,023 images (4 classes)	EfficientNetV2 + Vision Transformer ensemble
Hussain et al. (2026)	PMRAM dataset (4 classes)	CNN-LSTM + TSRF with PCA and RFE
Gómez-Guzmán et al. (2025)	Kaggle MRI dataset; 7,023 images	Swin Transformer (transfer learning)
Batool and Byun (2025)	Kaggle MRI dataset; 3,264 images	Lightweight multi-path CNN
Anand et al. (2026)	MRI dataset; 3,264 images	Optimized custom CNN
Proposed QGC-CNN	Kaggle dataset (~7,000 images; 4 classes)	QGC-CNN + Stacking Ensemble (ResNet50, Xception, EfficientNetB0) + LightGBM

**Table 2:** Performance comparison of existing methods along with identified limitations, illustrating the trade-offs between accuracy, complexity, and generalization.

References	Metrics	Results	Limitations	Problem solved
Lai et al. (2025)	Accuracy, Precision, Recall, F1, AUC	100% accuracy	High transformer complexity	Reduces computational cost
Maloji et al. (2024)	Accuracy, IoU	99.03% accuracy	Limited generalization	Improves early detection
Zarenia et al. (2025)	Accuracy, Precision, Recall	96.35% accuracy	Class imbalance, false positives	Improves interpretability
Heythem et al. (2024)	Accuracy, F1, MCC	99.76% accuracy	Limited dataset scope	Lightweight alternative
Tariq et al. (2025)	Accuracy, Precision, Recall	96% accuracy	Dataset inconsistency	Combines CNN + Transformer
Hussain et al. (2026)	Accuracy, F1, Kappa	Best performance among variants	Overfitting risk	Feature optimization + XAI
Gómez-Guzmán et al. (2025)	Accuracy, F1, MCC	99.24% accuracy	High complexity	Edge deployment feasibility
Batool and Byun (2025)	Accuracy, F1	96.03% accuracy	Noise sensitivity	Efficient multi-scale learning
Anand et al. (2026)	Accuracy, F1	98.5% accuracy	Noise sensitivity	Lightweight CNN design
Proposed QGC-CNN (stack ensemble)	Accuracy, F1, ROC-AUC, MCC, Log Loss, FLOPs	95.50%, AUC 0.99, 0.83 GFLOPs	Balancing efficiency vs global context	Efficient query-guided attention with calibrated predictions

As observed from Tables 1, 2 it was found that the current techniques have high accuracy in classification. However, there are some major drawbacks, such as high computational cost, inability to capture long-range dependencies, and poor probabilistic estimation. Among these techniques, though the performance of the hybrid model and the transformer-based model has been enhanced in terms of global feature representation, there is a major drawback of high computational cost. Similarly, though the performance of the ensemble-based model has been enhanced, there is a major drawback in efficiency and reliability in probability estimation. However, in the case of the proposed QGC-CNN framework, it was found that there is a better trade-off in terms of accuracy, efficiency, and interpretability. Moreover, the proposed framework has the ability to capture both local and global dependencies at a low computational cost. The performance of the framework has been enhanced through the stacking ensemble method with a LightGBM meta-learner.

In light of the research gaps identified, the proposed approach aims at efficiently extracting features, modeling the global context, and making use of ensemble learning for decision-making. Based on these identified limitations, the proposed methodology is designed to address the trade-off between accuracy, efficiency, and interpretability, which is discussed in subsequent section.

## 3 Materials and methods

**Figure 1:**
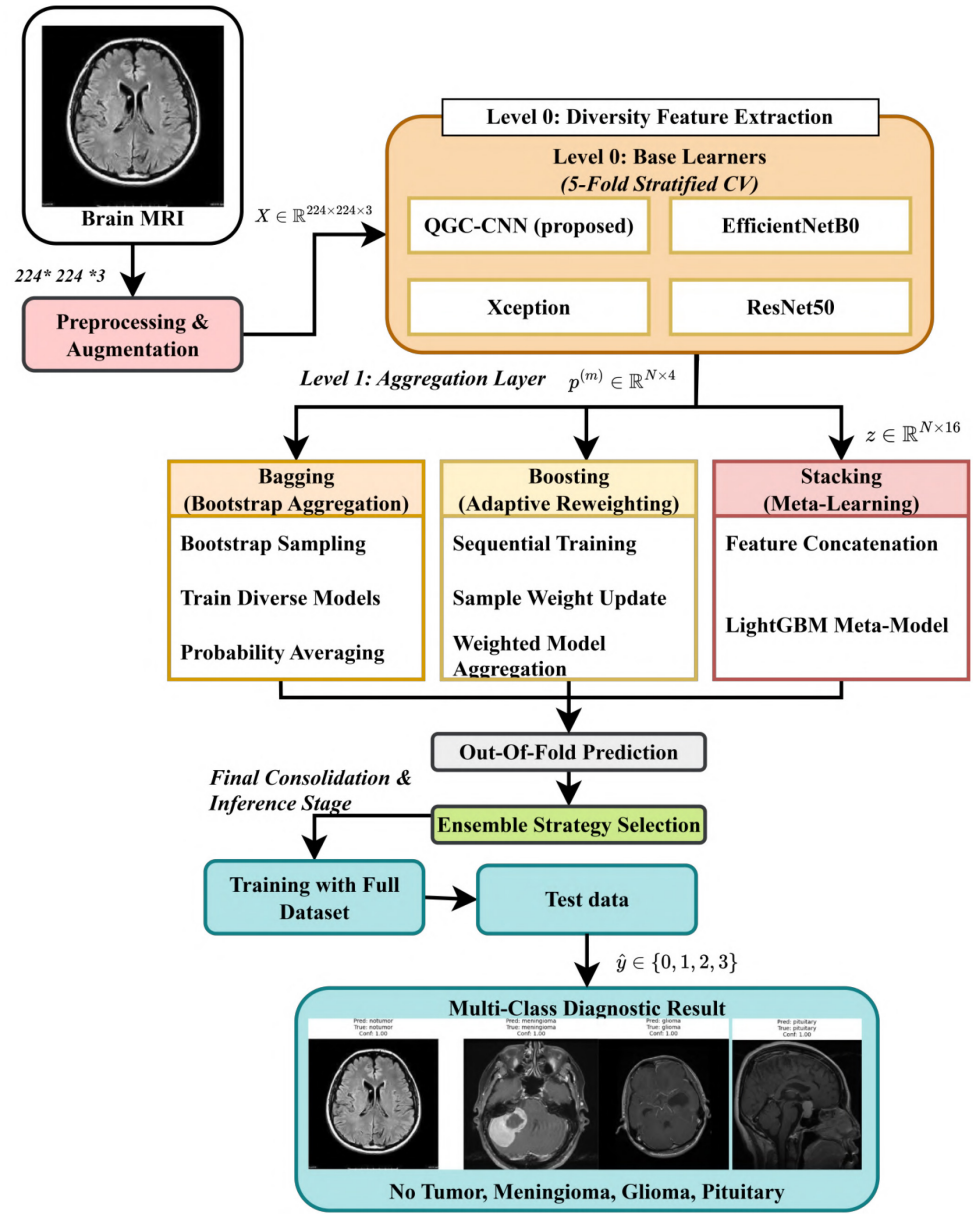
End-to-end workflow for multi-class brain tumor classification, illustrating the Level 0 feature extraction via four base learners and the Level 1 meta-learning fusion via LightGBM.

This study introduces a Query-Guided Cross-Attention Convolutional Neural Network (QGC-CNN) classifier with learnable query tokens and integrates it into a stacked ensemble framework with multiple strong CNN backbones and a Light Gradient Boosting Machine (LightGBM) meta-learner (Ke et al., 2017). The methodology is structured to maximize predictive accuracy by combining the strengths of diverse deep learning (DL) architectures. The model is executed using TensorFlow and Keras, for the base models and LightGBM for the meta-classifier. Before going into the architecture deeply, the complete work pipeline of this study is demonstrated with Figure 1.

### 3.1 Base models (level 0)

The first level of the ensemble consists of four distinct DL models. Training each of the selected DL models independently was the main focus. This was implemented using a five-fold cross-validation approach (Stone, 1974; Geisser, 1975; Kohavi, 1995; Hastie et al., 2009) to provide robust, out-of-fold predictions. The proposed framework initially focuses on extracting robust feature representations. This extraction was performed using well-known Convolutional Neural Network (CNN) architectures. CNN is a DL model designed for grid-like data such as images. It utilizes the convolutional filters to extract local features like edges, textures, and shapes. For an MRI scan, Equation 1,


$$\begin{array}{l} x\in {\mathbb{R}}^{224\times 224\times 3}, \end{array}$$ (1)

each CNN backbone produces a feature map as in Equation 2:


$$\begin{array}{l} F=f_{\text{CNN}}\left(x\right),\;F\in {\mathbb{R}}^{h\times w\times d}, \end{array}$$ (2)

where the symbols *h* and *w* denote the spatial dimensions of the feature map, and *d* indicates the depth (meaning, number of channels). For this study, three well-established CNN architectures were employed, which were pre-trained on the ImageNet dataset (Deng et al., 2009). The architectures were as follows:

- Xception: A model that relies on depthwise separable convolutions in order to create a highly efficient as well as a powerful feature extractor (Chollet, 2017).
- EfficientNetB0: It is a basic model of the EfficientNet family, which uniformly scales network breadth, depth, and resolution using a compound scaling technique (Tan and Le, 2019).
- ResNet50: A well-known as well as a traditional architecture that solved the vanishing gradient issue in very deep networks by including residual connections (He et al., 2016).

These three models were selected because they provide a diverse range of ensembles, as they leverage different strategies for feature extraction, which assists in reducing model variance and enhance generalization. For each of these models, the pre-trained backbone serves as a feature extractor. A new classification head is added, consisting of a GlobalAveragePooling2D layer (Lin et al., 2013) followed by a Dense layer with a softmax activation function for pituitary, meningioma, glioma, and no tumor four-class classification. While the base models capture strong feature representations, they remain limited in modeling long-range dependencies. To address this limitation, a QGC-CNN architecture is proposed.

### 3.2 Novel query-guided cross-attention CNN (QGC-CNN)

**Figure 2:**
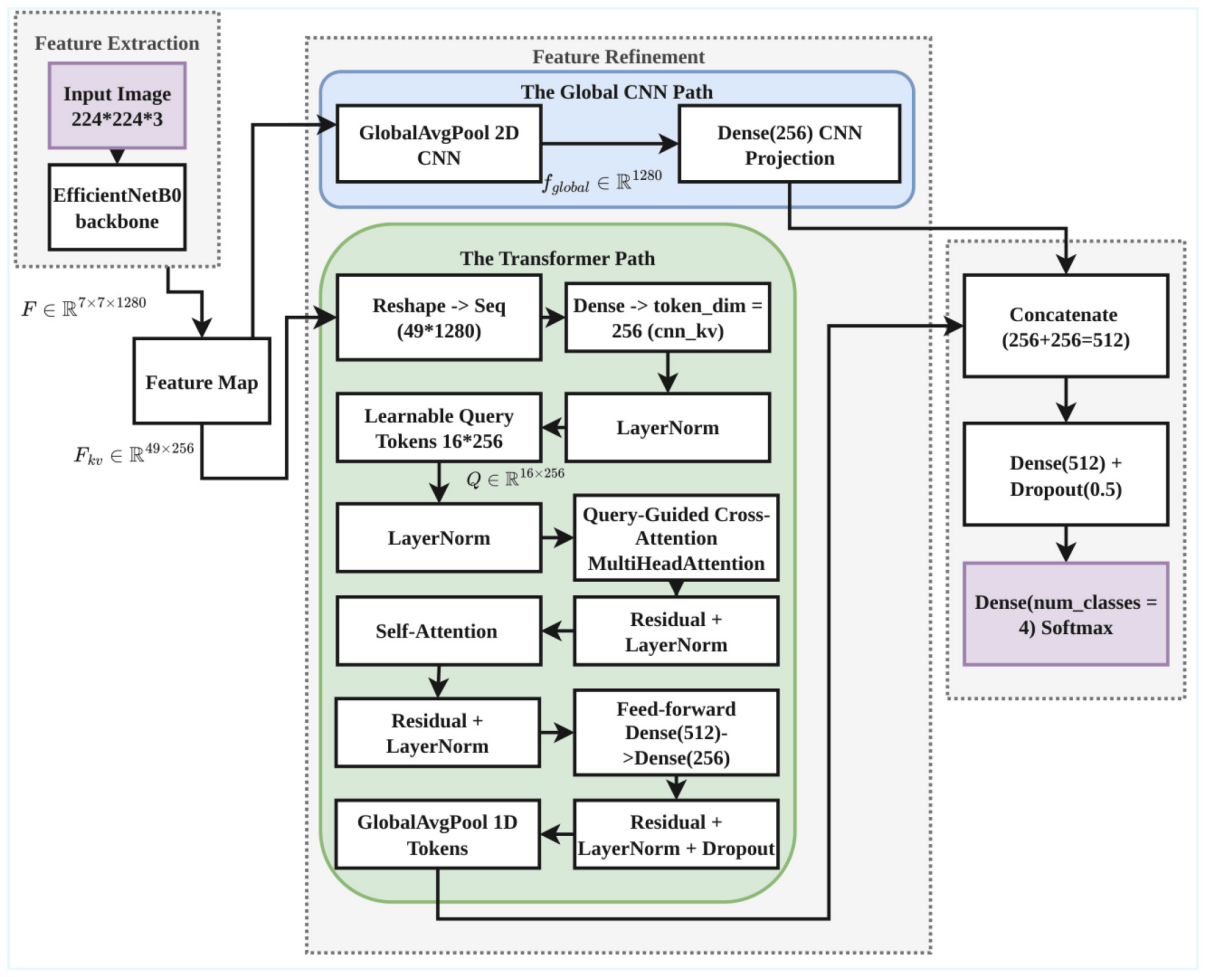
The Query-Guided Cross-Attention CNN (QGC-CNN) Architecture. The QGC-CNN employs EfficientNetB0 as its backbone for feature extraction. Then, it divides into a Global CNN branch and a Transformer branch where the latter takes learnable queries to extract long-term dependencies through cross-attention layers.

For the key contribution, a custom Query-Guided Cross-Attention CNN (QGC-CNN) model was built, which effectively integrates a CNN with a Transformer-based attention mechanism (Chen et al., 2021; Liu et al., 2021). The QGC-CNN architecture employing parallel CNN and attention-based pathways for enhanced medical image classification can be visualized in Figure 2.

This QGC-CNN architecture is designed to extract contextual information from MRI scans, both locally and globally. Though CNNs are most effective at learning local features (small patterns such as tumor textures), they fail to capture global context (relations between far-off parts of the image). Conversely, Transformers, which were designed for natural language processing tasks. These rely on attention mechanisms to capture long-range dependencies (Vaswani et al., 2017), making them powerful for images as well (Dosovitskiy, 2020). So, a hybrid model is designed where CNNs extract local features, and a transformer module captures global context via learnable query tokens (Carion et al., 2020). The processing pipeline of the proposed model is as follows. First, an input MRI scan is processed by EfficientNetB0 (ImageNet-pre-trained, top removed) (Tan and Le, 2019). The model is supplied with a (224, 224, 3) input image and the backbone outputs a feature map of shape (7, 7, 1280). This map is reshaped into a sequence of 49 patches and linearly projected to a lower dimension of 256. A small set of **learnable query tokens** (*k* = 16, *d* = 256) then interact with these features through a cross-attention mechanism, empowering the model to selectively focus on regions (Carion et al., 2020) relevant for tumor classification. The attended token embeddings are aggregated and concatenated with the CNN's global average features. The fused representation is fed to a fully connected softmax classifier to output tumor probabilities. The list of parameters used for this model is presented in Table 3.

**Table 3:** Comprehensive specifications of the proposed Query-Guided Cross-Attention CNN (QGC-CNN).

Component	Configuration
CNN backbone	EfficientNetB0 (ImageNet pre-trained, top removed)
Feature projection	Dense layer → 256-dim patch embeddings
Query tokens	*k* = 16, *d* = 256
Transformer	1 block, 8 heads, FFN = 512, dropout = 0.1
Classifier	Concatenation (tokens + CNN global avg), Dense softmax

Comprehensive specifications of the proposed Query-Guided Cross-Attention CNN (QGC-CNN).

#### 3.2.1 Learnable query tokens

The architecture incorporates a proposed LearnableQueryTokens layer, which is structured as a trainable, randomly initialized embedding layer. These are basically trainable vectors that act like “virtual questions” posed to the feature map, guiding the Transformer to search for specific abnormal regions in the MRI. These query tokens are designed to learn to extract and attend to the most informative aspects of the CNN feature map for the classification task. Cross-attention allows these queries to attend to important areas, mimicking how radiologists scan an image for anomalies. This approach, where learnable queries interact with image features, was popularized by models in object detection (Carion et al., 2020). Formally, the query tokens are represented as in Equation 3:


$$\begin{array}{l} Q\in {\mathbb{R}}^{k\times d}, \end{array}$$ (3)

where *k* represents the number of tokens and *d* indicates token dimension. They learn to focus on specific aspects of tumor features across all images.

#### 3.2.2 Cross-attention

To enable interaction between query tokens and image features, a cross-attention mechanism is employed. A MultiHeadAttention layer is applied to carry out cross-attention. The learnable query tokens are the “query,” and the projected CNN features are both the “key” and “value.” This helps the model identify the most important portions of the image for classification. Queries interact with CNN features *F* via the scalable dot-product attention mechanism (Vaswani et al., 2017) as in Equation 4:


$$\begin{array}{l} \text{Attention}\left(Q,F\right)=\text{softmax}\left(\frac{QW_{Q}{\left(FW_{K}\right)}^{⊤}}{\sqrt{d}}\right)FW_{V}, \end{array}$$ (4)

where *W*_*Q*_, *W*_*K*_, *W*_*V*_ are trainable projection matrices. Intuitively, this lets each query token attend to various regions of the image which is being passed, similar to radiologists scanning an MRI slice for abnormalities. Following attention, the extracted representations are fused to form a unified feature vector.

#### 3.2.3 Token fusion

All attended query embeddings are pooled and concatenated with CNN global averages through Equation 5:


$$\begin{array}{l} z=\text{AvgPool}\left(\text{Attention}\left(Q,F_{kv}\right)\right)⊕\text{GlobalAvgPool}\left(F\right), \end{array}$$ (5)

where ⊕ denotes concatenation. This reassures both local as well as global information is preserved for classification. Finally, the fused representation is used for classification.

#### 3.2.4 Classification head

A very significant feature for classification tasks, which was a dense (fully connected) layer with softmax outputs the final class probabilities for tumor types. Architecturally, the output tokens obtained from the Transformer block are flattened. Separately, the original CNN feature map is passed through a GlobalAveragePooling2D layer (Lin et al., 2013). The flattened token features as well as the pooled CNN features are then concatenated. The anticipated class probabilities are then generated by feeding this integrated feature vector into a final Dense layer with a softmax activation function. The entire pipeline of the model is explained by Algorithm 1.

Algorithm 1 Query-Guided Cross-Attention CNN (QGC-CNN). 
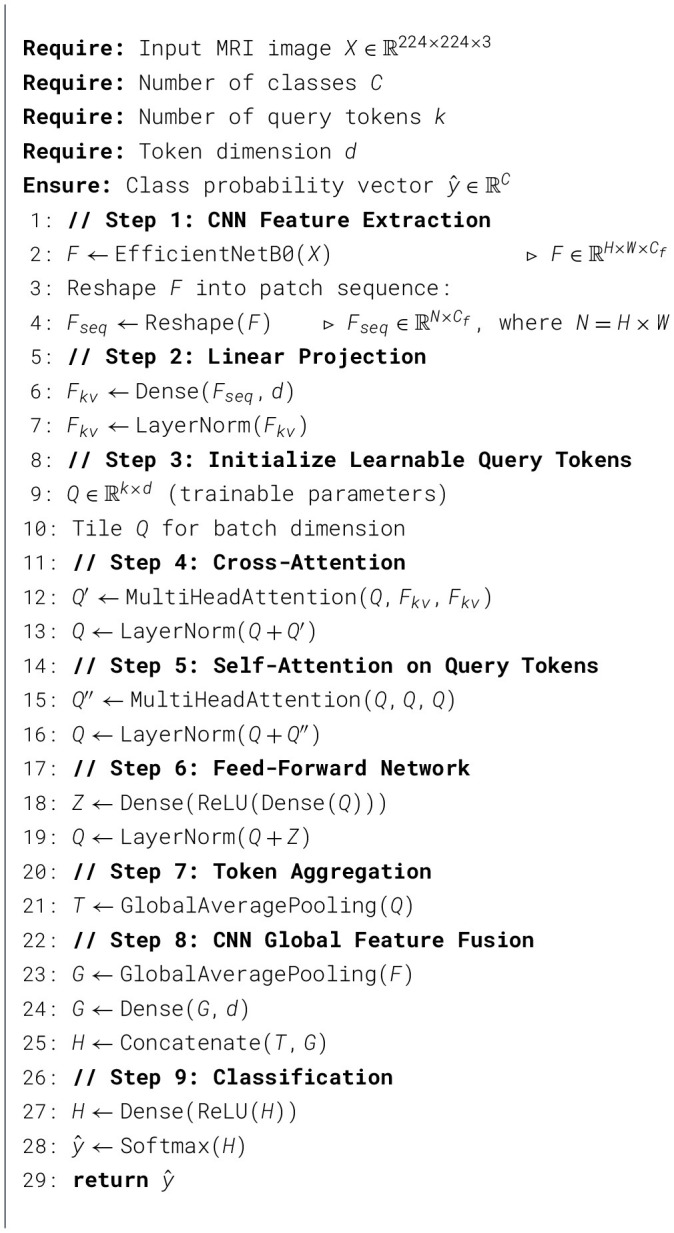

To improve the performance and to make the model reproducible, the corresponding hyperparameters were optimized utilizing the five-fold cross-validation technique. In light of the considerable resources needed for training deep neural network models, a thorough optimization procedure including automated searches such as grid or Bayesian approaches were avoided in favor of heuristic manual optimization. The initial value for hyperparameters like learning rate, batch size, dropout rate, as well as the size of the transformers was determined using previous work regarding ResNets, EfficientNets, and Transformers' benchmarks. This information was then further tuned according to how well the models performed during validation within cross-validation folds. In order to avoid any problems associated with overfitting, an early stopping procedure was employed (Prechelt, 1998) in addition to employing a dynamic learning rate scheduler in the form of ReduceLROnPlateau, decreasing it if there was no improvement in the validation loss metric. Lastly, the optimal epoch number was estimated as an average of convergence point for all folds. The specific hyperparameter values and final configurations are detailed in the Experimental Results section.

### 3.3 Ensemble strategies

While the proposed QGC-CNN provides strong feature representations, further improvements can be achieved by combining multiple models through ensemble learning. A machine learning technique known as ensemble learning (Yang et al., 2023; Sagi and Rokach, 2018) aims to increase a model's forecast accuracy by mixing the predictions of several base models rather than relying only on one. The basic idea would be, multiple models may learn different aspects of the data distribution, so combining these models can help in reducing the bias as well as variance of the predictions. In medical image analysis, ensemble methods can help in improving the robustness of the model. Three complementary ensemble approaches are investigated, applied over the same set of four base learners (Xception, EfficientNetB0, ResNet50, and the proposed QGC-CNN): (i) stacking, (ii) bagging (Breiman, 1996), and (iii) boosting (Freund and Schapire, 1997), (Schapire, 2013). The predictions or outcomes of the base models are combined in a distinct manner by each strategy. In the stacking approach, a meta-learner is employed to learn an optimal combination of the base-level predictions. Bagging reduces variance by aggregating predictions from models trained on bootstrap-resampled datasets. Boosting sequentially refines model training by adaptively emphasizing misclassified samples. By evaluating these ensemble paradigms within a unified experimental framework, a systematic analyzes on how different aggregation strategies influence classification performance for multi-class brain tumor diagnosis is studied. The following subsections describe each ensemble method in detail.

#### 3.3.1 Stacked ensemble meta-model (Level 1)

Stacked generalization (stacking) is one of the well-established ensemble strategy that combines multiple base learners through a higher-level meta-model (Wolpert, 1992). Unlike simple averaging, stacking learns how to optimally weight and combine the predictions of heterogeneous models. In this study, LightGBM (Light Gradient Boosting Machine) (Ke et al., 2017) is employed as the meta-learner at Level 1. Let *p*^(*m*)^∈ℝ^*C*^ denote the class probability vector produced by the *m*-th base model, where *C* = 4 defines the number of tumor classes and *m*∈{Xception, EfficientNetB0, ResNet50, QGC-CNN}. The meta-feature vector for a single sample is constructed by concatenating the outputs of all base learners (Equation 6):


$$\begin{array}{l} \text{z}=\left[p^{\left(\text{Xception}\right)},p^{\left(\text{EfficientNetB0}\right)},p^{\left(\text{ResNet50}\right)},p^{\left(\text{QGC-CNN}\right)}\right]\in {\mathbb{R}}^{4C}. \end{array}$$ (6)

Since *C* = 4, the resulting meta-feature vector would have the dimensionality of 16. The final prediction is obtained by passing this vector into LightGBM. The final prediction is obtained as Equation 7:


$$\begin{array}{l} ŷ=\text{LightGBM}\left(\text{z}\right). \end{array}$$ (7)

1. **Meta-feature construction (out-of-fold strategy):** The meta-model of this work is trained using out-of-fold (OOF) predictions generated via five-fold cross-validation to avoid information leakage and also to ensure unbiased training. For every training sample, the probability vector used as a meta-feature is extracted from a base model that was not trained on the same fold as the current sample. This yields a meta-training dataset of size (*N*, 16), where *N* defines the count of training samples.
2. **Meta-learner selection:** The meta-learner was selected to be LightGBM because gradient boosting systems are specifically well-suited for handling tabular meta-features with non-linear relationships. Because the meta-features are probabilistic outcomes of heterogeneous deep models, a boosting algorithm based on a tree structure can capture complex relationships between models.
3. **Hyperparameter optimization:** The number of leaves, learning rate, and number of boosting iterations were optimized using a validation split with early stopping to avoid overfitting.
4. **Inference procedure:** While testing, each of the four base models generates a class probability vector for a given image. Because each base learner is trained under five-fold cross-validation, predictions from the five trained instances of each model are averaged to obtain a stable probability estimate (Equation 8):

$$\begin{array}{l} {\bar{p}}^{\left(m\right)}=\frac{1}{K}\overset{K}{\underset{k=1}{\sum }}p_{k}^{\left(m\right)},\;K=5. \end{array}$$ (8)

The averaged probability vectors from all base models are then concatenated to form the test meta-feature vector (Equation 9):


$$\begin{array}{l} {\text{z}}_{\text{test}}=\left[{\bar{p}}^{\left(\text{Xception}\right)},{\bar{p}}^{\left(\text{EfficientNetB0}\right)},{\bar{p}}^{\left(\text{ResNet50}\right)},{\bar{p}}^{\left(\text{QGC-CNN}\right)}\right]. \end{array}$$ (9)

This vector is finally passed through the trained LightGBM model to produce the final tumor classification.

In addition to stacking, alternative ensemble strategies such as bagging and boosting are also explored to provide a comprehensive evaluation.

#### 3.3.2 Multi-architecture bagging strategy

Bootstrap Aggregating (Bagging) is an ensemble learning strategy that can help alleviate model variance by teaching multiple models on resampled versions of the training set and then aggregating their predictions or outcomes (Breiman, 1996). The classic bagging strategy involves training identical models on distinct bootstrap samples. In this paper, the strategy is generalized to a heterogeneous environment where each model can be selected in a cyclic manner from a heterogeneous architecture pool including Xception, EfficientNetB0, ResNet50, and QGC-CNN. Let $D={\left\{\left(x_{i},y_{i}\right)\right\}}_{i=1}^{N}$ denote the training dataset. For each bagging iteration *b* = 1, …, *B*, a bootstrap dataset $D_{b}$ is generated by sampling with replacement from D. A base learner *f*_*b*_(·) is then trained on $D_{b}$. Unlike conventional bagging, the base learner at each iteration is selected from a pool of diverse architectures:


$$f_{b}\in \left\{\text{Xception},\text{EfficientNetB0},\text{ResNet50},\text{QGC-CNN}\right\}.$$

This brings in architectural diversity, apart from the existing data diversity. The ultimate prediction is made by averaging the predicted probabilities for all models as described in Equation 10.


$$\begin{array}{l} ŷ=\arg\underset{c}{\max}\left(\frac{1}{B}\overset{B}{\underset{b=1}{\sum }}p_{b}\left(c|x\right)\right), \end{array}$$ (10)

where *p*_*b*_(*c*∣*x*) represents the predicted probability of class *c* from the *b*-th model. This assist to mitigate the impact of data variability by integrating the benefits of bootstrap resampling and also multiple architectures. To facilitate a strong evaluation, Bagging with a 5-fold cross-validation strategy (Stone, 1974; Geisser, 1975; Kohavi, 1995; Hastie et al., 2009) is combined. In each iteration of the cross-validation, *N*_*bags*_ = 4 bootstrap samples are created for each of the four architectures, leading to an ensemble of 80 models. This two-level diversity (data level and architecture level) helps to greatly reduce the variance of the tumor-specific representations.

#### 3.3.3 Multi-architecture boosting strategy

Boosting is an iterative ensemble learning strategy that allows models to be trained using samples that were wrongly classified in earlier rounds (Freund and Schapire, 1997; Schapire, 2013). Unlike bagging, boosting adds a dependency between models by means of a reweighting process. Let $w_{i}^{\left(t\right)}$ denote the weight assigned to training sample *i* at boosting iteration *t*. Initially, all samples are assigned equal weights (Equation 11):


$$\begin{array}{l} w_{i}^{\left(1\right)}=\frac{1}{N},\;i=1,\ldots ,N. \end{array}$$ (11)

In each boosting iteration *t*, we seek to optimize a sub-model *h*_*t*_(·) from a diverse set of architectural candidates P={Xception, EfficientNetB0, ResNet50, QGC-CNN} with the goal of minimizing the distribution-weighted error ε_*t*_ given by Equation 12:


$$\begin{array}{l} \varepsilon_{t}=\overset{N}{\underset{i=1}{\sum }}D_{t}\left(i\right)\cdot 1_{h_{t}\left(x_{i}\right)\neq y_{i}} \end{array}$$ (12)

where *D*_*t*_(*i*) is the probability distribution over the training data, and **1** is the identity indicator for classification error. The contribution coefficient β_*t*_ for the chosen sub-model is then obtained through Equation 13:


$$\begin{array}{l} \beta_{t}=\frac{1}{2}\ln\;\left(\frac{1-\varepsilon_{t}}{\varepsilon_{t}}\right) \end{array}$$ (13)

To focus on the more challenging tumor samples in the next iteration, the weights for importance are updated using an exponential formula Equation 14:


$$\begin{array}{l} D_{t+1}\left(i\right)=\frac{D_{t}\left(i\right)\exp\left(\beta_{t}\cdot 1_{h_{t}\left(x_{i}\right)\neq y_{i}}\right)}{Z_{t}} \end{array}$$ (14)

In this expression, *Z*_*t*_ serves as a partition function to ensure that *D*_*t*+1_ remains a valid probability distribution (∑*D*_*t*+1_ = 1).

To begin with, all samples are assigned the same weight. In each round, a model is trained on the weighted set of samples, selected from the same set of diverse architectures (Xception, EfficientNetB0, ResNet50, and QGC-CNN). In our implementation, each individual base learner *m* is given a weight α_*m*_ according to its weighted error rate ϵ_*m*_. In each boosting iteration *t*, the weights of the samples *w* are updated to give higher priority to the samples that are incorrectly categorized in the prior iteration in the next sampling step: *w*_*t*+1_ = *w*_*t*_·exp(α_*t*_·*I*), where *I* is the indicator function for misclassification. This helps to ensure that the ensemble learns to correctly classify the “hard” samples, such as the boundaries of Meningioma tumors. The classification error is calculated based on the weights of the samples, and each model is assigned a weight according to its performance. In the subsequent round, samples that were incorrectly classified are given higher weights, guaranteeing that the subsequent model will give them more consideration. The weighted forecasts from each model are added together to produce the final forecast, as described in Equation 15.


$$\begin{array}{l} ŷ=\arg\underset{c}{\max}\left(\overset{T}{\underset{t=1}{\sum }}\alpha_{t}p_{t}\left(c|x\right)\right). \end{array}$$ (15)

As the robustness of adaptive reweighting and architectural diversity integrated, this boosting method is able to sharpen decision boundaries iteratively and capitalize on the complementary qualities of various other CNN architectures.

#### 3.3.4 Comparative evaluation of ensemble paradigms

It is significant to mention that the evaluation of stacking, bagging, and boosting is conducted as separate ensemble paradigms in the same setting. Each approach is applied on the same set of four base learners (Xception, EfficientNetB0, ResNet50, and QGC-CNN) to make a fair comparison. The aim is not to make a combination of all three ensemble approaches at once but to examine the impact of different aggregation principles on the multi-class brain tumor classification task. To ensure fair comparison and avoid information leakage, the performance of each ensemble paradigm (stacking, bagging, and boosting) was first evaluated using five-fold out-of-fold (OOF) predictions on the training dataset. Based on the OOF accuracy and macro-F1 score, the best-performing ensemble strategy was selected. The selected ensemble was then retrained using the entire training dataset, without cross-validation splits, to maximize the use of available data. Finally, the retrained model was evaluated once on the independent test set to report the final performance metrics.

### 3.4 Explainability and representation analysis

Beyond predictive performance, it is essential to understand the internal decision-making behavior of the proposed models. To explore the internal learning behavior of the proposed QGC-CNN model and baseline architectures (ResNet50, Xception, and EfficientNetB0), multiple complementary interpretability strategies were employed.

#### 3.4.1 Grad-CAM visualization for baseline CNNs

Deep learning models are often treated as “black boxes.” To ensure transparency Gradient-weighted Class Activation Mapping (Grad-CAM) (Selvaraju et al., 2017) is integrated. This approach produces a localization map *M*^*c*^∈ℝ^*u*×*v*^ for any target category *c*. First, the importance weights $\omega_{k}^{c}$ for each feature map *A*^*k*^ are obtained by taking the global average pooling of the gradients of the class score *y*^*c*^ with respect to the convolutional activations, Equation 16:


$$\begin{array}{l} \omega_{k}^{c}=\frac{1}{Z}\underset{i}{\sum }\underset{j}{\sum }\frac{\partial y^{c}}{\partial A_{i,j}^{k}} \end{array}$$ (16)

Next, the class-specific heatmap is obtained by taking a weighted linear combination of the forward-pass activations, followed by a Rectified Linear Unit (ReLU) activation to remove features with non-positive impact on the target class (Equation 17):


$$\begin{array}{l} M^{c}=\text{ReLU}\left(\underset{k}{\sum }\omega_{k}^{c}A^{k}\right) \end{array}$$ (17)

Before global pooling, the last convolutional layer was subjected to Grad-CAM. The class-specific gradient was computed with respect to the predicted category, and channel-wise importance weights were obtained by global average pooling of gradients. The resulting activation map was normalized and superimposed onto the original MRI slice to highlight discriminative regions influencing the prediction. This procedure enables spatial localization of tumor-relevant regions learned by standard convolutional architectures.

#### 3.4.2 Hierarchical feature extraction in QGC-CNN model

To further analyze the attention mechanism introduced in QGC-CNN, a dedicated query-guided attention analysis is performed. For the proposed QGC-CNN architecture, intermediate feature activations were extracted from four stages of the EfficientNetB0 backbone as follows:

- block2a_expand_activation (early edge-level features)
- block4a_expand_activation (mid-level textures)
- block6a_expand_activation (high-level tumor structures)
- top_activation (semantic representation)

For each stage, feature maps were averaged across channels to generate spatial activation summaries. Additionally, quantitative metrics such as Mean absolute activation intensity, Maximum activation magnitude, and Feature sparsity (percentage of near-zero activations) were computed. The result of these metrics provide insight into how feature abstraction evolves across the depth of network.

### 3.5 Query-guided cross-attention analysis

The proposed model introduces 16 learnable query tokens that attend to CNN-derived patch embeddings via multi-head cross-attention. To analyze this mechanism: Normalized query and key-value tensors were extracted. Scaled dot-product attention weights were computed. Attention maps were reshaped to spatial resolution and overlaid on MRI images. For quantitative characterization, the following metrics were calculated using Peak attention focus (maximum attention weight per token), Token focus concentration (coefficient of variation of attention distribution), These metrics evaluate how selectively each query token attends to spatial tumor regions.

### 3.6 Latent representation analysis

To assess class separability in the learned representation space: The 512-dimensional feature vector (pre-softmax) was extracted. Features were standardized. t-SNE was applied for 2D visualization (van der Maaten and Hinton, 2008). Silhouette score was computed to measure clustering quality (Rousseeuw, 1987). Intra-class variance and inter-class centroid distance were calculated. These metrics quantify how effectively each architecture organizes tumor classes in latent space.

### 3.7 Learning efficiency comparison

Intermediate mid-to-late stage activations were extracted from each model and visualized as spatial heatmaps. This comparison illustrates how different architectures progressively refine tumor-related patterns. The methodological framework explained detailed flow of our system. Following the methodological and analytical framework, the experimental results are presented in the next section.

## 4 Results

This section deals with the experimental validation of the proposed framework, namely, the QGC-CNN, along with the ensemble schemes. The analysis of the framework is conducted in terms of classification, computational efficiency, and reliability with respect to various metrics. First, the dataset, along with the preprocessing, is discussed, followed by the experimental setup, metrics, and then the analysis of individual models, followed by ensemble models using cross-validation, OOF, class-wise, and complexity-based analysis.

### 4.1 Dataset

The dataset utilized in this study is publicly available Brain Tumor MRI dataset hosted on Kaggle (Nickparvar, 2021). The dataset comprises about 7,200 images divided into four classes: Glioma, Meningioma, Pituitary tumor, and No tumor, with an equal number of images (1,800 each) per category. The class-wise distribution of the dataset is summarized in Table 4.

**Table 4:** Class-wise distribution of the Brain Tumor MRI dataset.

Class	Training samples	Testing samples	Total samples
Glioma	1,400	400	1,800
Meningioma	1,400	400	1,800
Pituitary tumor	1,400	400	1,800
No tumor	1,400	400	1,800
Total	5,600	1,600	7,200

As observed, this balanced distribution ensures that the model is not biased toward any specific class and allows for fair evaluation across all tumor categories. It is important to note that this dataset is a carefully curated collection from several freely available datasets, namely the Figshare brain MRI dataset (Cheng et al., 2015), the SARTAJ dataset (Sartaj, 2020), and the Br35H dataset (Hamada, 2020). The dataset combines samples from multiple sources, which may introduce variation in the data and support evaluation of model robustness. Despite being provided with an initial train-test split (5,600 training images and 1,600 testing images), the dataset lacks patient-level information for separating images by subjects. To maintain a coherent and reliable evaluation process, all training set images have been merged and a five-fold Stratified Cross-Validation scheme was performed on an image basis. Stratified Cross-Validation allows class balance in all folds and full dataset usage. Within each fold, data augmentation techniques were only applied to the training part randomly applying rotations (±20°), translations, zooms, and horizontal flips. No data augmentation was applied to validation/test partitions in order to keep them untouched. As it should be highlighted, without patient-level identifiers, it was impossible to strictly divide the images by their owners (patients). No direct data leakage was observed in terms of duplicated samples across splits; however, due to the absence of patient-level identifiers, potential inter-sample correlations cannot be completely ruled out. In the lack of identification information from individual patients, a careful approach should be taken toward understanding internal measures. Although our Out-of-Focus approach stops us from directly replicating any images, the issue of 'slice level leakage' still poses a problem toward clinical applicability. In order to resolve this, the model's capacity to properly diagnose is evaluated not only by its performance on internal testing but also on an external independent dataset.

#### 4.1.1 Dataset pre-processing

- Preprocessing: All images resized to 224 × 224, pixel intensities normalized to [0,1].
- Augmentation: Rotation (±20°), width/height shifts (20%), zoom (20%), horizontal flips.
- Rationale: Data augmentation prevents overfitting and simulates real-world imaging variations.

Additionally, the stacking ensemble (will be discussed in subsequent section) is made with out-of-fold (OOF) predictions, wherein the base models make predictions for the validation sample alone. Thus, the training of the meta-model (LightGBM) occurs on predictions of completely independent samples, thereby avoiding any kind of indirect data leakage. To address concerns regarding dataset diversity and the ability of the proposed model to generalize well, the proposed approach is tested on a completely new external dataset, the Mendeley Brain Tumor MRI Dataset (Hira et al., 2026), comprising images of four classes. To enhance the utility of the system out of the box, the architecture was tested on a pre-processing pipeline of the minimal kind. Unlike other approaches, which need exact skull stripping (an inherently error-prone method in practice), the proposed QGC-CNN makes use of its built-in attention component to extract pathogenic features of interest from the anatomical background. The robust performance across multiple institutions despite varying normalization techniques reinforces this point.

#### 4.1.2 Experimental setup

- Hardware: NVIDIA RTX GPU (12 GB VRAM), 64 GB RAM.
- Software: TensorFlow 2.13, Keras, LightGBM, Python 3.10.
- Optimizer: Adam with learning rate η = 10^−4^.
- Batch size: 32, Epochs: 50, early stopping patience = 5.
- Cross-validation: A StratifiedKFold strategy with 5 splits (n_splits = 5) was employed. This ensures that the class distribution in each fold is representative of the overall dataset distribution.
  The data was shuffled before splitting (shuffle = True) with a fixed random state (random_state = 42) for reproducibility.
- Loss function: To supervise the multi-class learning process, a Categorical Cross-Entropy (CCE) is used. This loss measures the difference between the ground truth distribution and the model's Softmax outputs. Formally, for *N* observations over *C* categories, the cost *L* is calculated as in Equation 18:
$$\begin{array}{c} L\left(\theta \right)=-\frac{1}{n}\overset{n}{\underset{i=1}{\sum }}\overset{K}{\underset{k=1}{\sum }}1_{y_{i}=k}\ln\left(p_{i,k}\right) \end{array}$$ (18)
  where **1**_*y*_*i*_ = *k*_ represents the indicator function for the target class *k*, and *p*_*i, k*_ denotes the predicted probability for the *i*-th instance belonging to category *k*.
- ReduceLROnPlateau: The learning rate was reduced by 0.2 if, after two successive epochs (patience = 2), the validation loss did not improve.

To quantitatively evaluate model performance, a set of standard classification and efficiency metrics is employed in the following subsection.

### 4.2 Performance metrics

To fairly assess the proposed system, standard performance metrics as well as efficiency measures are adopted. All classification metrics were computed using macro-averaging across classes to ensure equal contribution from each tumor category. The symbols *TP*, *TN*, *FP*, and *FN* represent True Positives, True Negatives, False Positives, and False Negatives, respectively, as obtained from the class-wise confusion matrix. The metrics Equations 19–24, and the definitions were as follows:

1. Accuracy: Represents the global ratio of correct predictions:
$$\begin{array}{c} \text{Accuracy}=\frac{TP+TN}{TP+TN+FP+FN} \end{array}$$ (19)
2. Precision: It evaluates the proportion of correctly predicted positive samples among all predicted positives:
$$\begin{array}{c} \text{Precision}=\frac{TP}{TP+FP} \end{array}$$ (20)
3. Recall (Sensitivity): This measures the proportion of correctly predicted positive samples among all actual positives:
$$\begin{array}{c} \text{Recall}=\frac{TP}{TP+FN} \end{array}$$ (21)
4. F1-score: A unified metric that harmonizes Precision and Recall:
$$\begin{array}{c} \text{F1-score}=2\cdot \frac{\text{Precision}\cdot \text{Recall}}{\text{Precision}+\text{Recall}} \end{array}$$ (22)
5. ROC-AUC (Receiver Operating Characteristic—Area Under Curve): The likelihood that a randomly selected positive instance would be ranked higher by the classifier than a negative instance is measured by ROC-AUC. With macro averaging, the One-vs.-Rest technique was used to calculate ROC-AUC for the multi-class context. It is computed from the ROC curve (TPR vs. FPR):
$$\begin{array}{c} \text{AUC}=\overset{1}{\underset{0}{\int }}TPR\left(FPR\right)d\left(FPR\right) \end{array}$$ (23)
  where
$$\begin{array}{c} TPR=\frac{TP}{TP+FN},\;FPR=\frac{FP}{FP+TN} \end{array}$$ (24)
6. Efficiency metrics: Alongside accuracy-based measures, computational efficiency is critical:
  - FLOPs (Floating-point operations): proxy for computational complexity.
  - Inference Time (*T*_*inf*_): average time to predict one sample.

### 4.3 Training result

The performance of the individual base learners on the five-fold cross-validation is summarized in Table 5. Although high-capacity models such as Xception and ResNet50 reported the highest absolute accuracy values of 98.70% and 98.29%, respectively, they did so at the expense of a high computational cost of 9.11 and 7.73 GFLOPs, respectively. In contrast, the proposed QGC-CNN obtained a competitive accuracy of 96.96%, thus closing the performance gap between light-weight baselines and heavy-duty models. Most importantly, the QGC-CNN reported an extremely low computational cost of 0.83 GFLOPs, which is approximately 10.9 times more efficient than Xception, with only a 1.74% difference in accuracy. However, by incorporating our Query-Guided Cross-Attention module, the Hybrid model (based on the same EfficientNetB0 architecture) not only reported an accuracy improvement of over 14% but also reported a significantly higher stability of ± 1.16%. This indicates that the learnable query tokens in our QGC-CNN module are able to capture global dependencies that are missed by the standard convolutional layers, thus providing a robust yet mobile-friendly solution for clinical brain tumor diagnosis.

**Table 5:** Comparative performance and computational complexity of level-0 base learners.

Metric	EfficientNetB0	QGC-CNN (Ours)	ResNet50	Xception
Params (M)	~5.3	~10.3	~25.6	~22.9
GFLOPs	0.79	0.83	7.73	9.11
Time (s/batch)	0.3101	0.3364	0.2841	0.2800
Accuracy (Mean ± SD)	82.36% ± 1.2%	96.96% ± 1.16%	98.29% ± 0.38%	98.70% ± 0.18%

**Figure 3:**
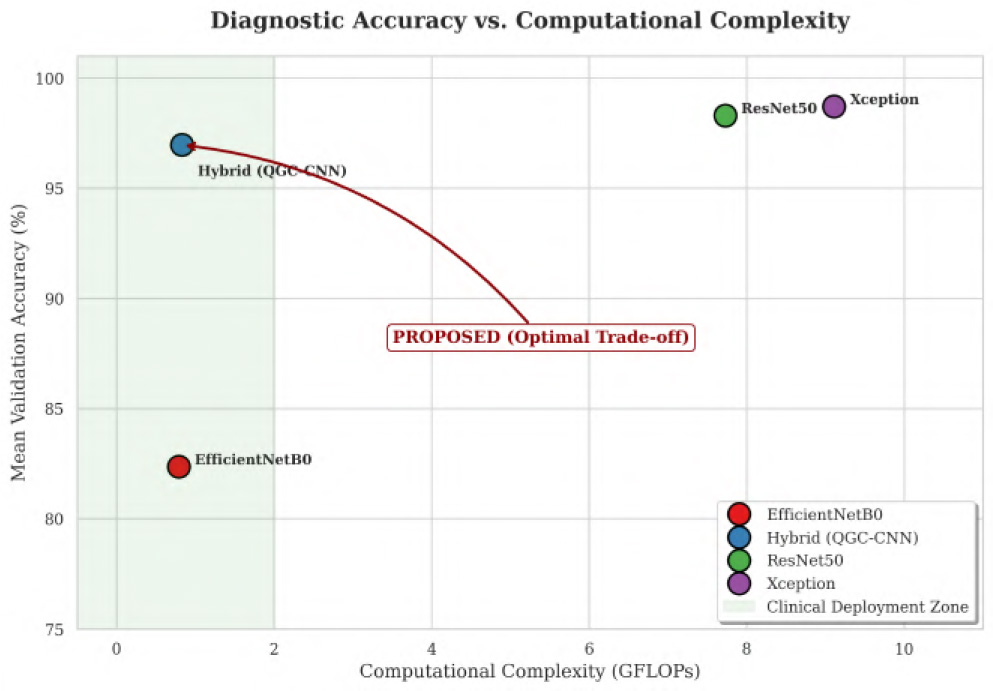
The proposed Hybrid (QGC-CNN) occupies the optimal “sweet spot,” providing Xception-level performance with a computational footprint similar to EfficientNetB0.

The relationship between model complexity (in GFLOPs) and the accuracy on the classification task is shown in Figure 3. The standard high-capacity models such as ResNet50 and Xception have strong diagnostic performance but are computationally expensive, with a complexity of over 7.5 GFLOPs. On the other hand, one of the baseline models, EfficientNetB0 is computationally efficient but has lower accuracy and higher variance between folds.

The proposed QGC-CNN occupies an intermediate position in this accuracy-complexity trade-off. With the aid of our Query-Guided Cross-Attention module, the model is able to attain a validation accuracy of 96.96% while being computationally efficient at 0.83 GFLOPs. This is a **10.9-fold reduction in complexity** over Xception with a mere accuracy difference of 1.74%. These findings clearly suggest that our learnable query tokens are able to capture high-level semantic representations of the input data without the need for the usual depth of standard convolutional networks, making the system potential for deployment.

In order to ensure a rigorous and well-rounded performance evaluation of all four tumor types, a comprehensive set of metrics was computed using the Out-of-Fold (OOF) prediction strategy. Macro-averaged Precision, Recall, and F1-score are used to avoid the dominance of performance evaluation by the majority class. In addition, clinical reliability was also measured using Macro Specificity, Cohen's Kappa, and Matthews Correlation Coefficient (MCC) (Matthews, 1975). The discriminative threshold was also measured using Macro ROC-AUC and Log Loss. Macro-averaged metrics were adopted to ensure balanced evaluation across all four tumor classes, preventing dominance by any single class. The overall performances of individual architectures on five-fold CV are represented in Table 6.

**Table 6:** Out-of-fold (OOF) performance metrics for all individual models.

Metric	QGC-CNN	Xception	ResNet50	EffNetB0
OOF accuracy	0.9691	0.9857	0.9817	0.8205
OOF macro F1	0.9690	0.9857	0.9817	0.8274
Macro recall	0.9691	0.9857	0.9817	0.8274
Macro specificity	0.9897	0.9952	0.993	0.9401
Cohen's Kappa	0.9588	0.9810	0.9757	0.7607
MCC	0.9592	0.9810	0.9758	0.7701
Macro ROC-AUC	0.9988	0.9995	0.99918	0.9791
Log loss	0.0959	0.0523	0.07	0.3612

Among the architectures, Xception and ResNet50 reported the best results in overall OOF accuracy at 98.57% and 98.17%, respectively. The proposed QGC-CNN model reported a very competitive outcome with an accuracy of 96.91% and MCC of 0.9592, which signifies a good level of correlation between the predicted and actual labels. Though EfficientNetB0 reported a lower accuracy (82.05%), it is worth noting that QGC-CNN reported state-of-the-art results at a much lower computational complexity (FLOPs) compared to the baseline Xception model. A detailed class-wise breakdown can be found in Table 7.

**Table 7:** Comprehensive per-class OOF metrics across all architectures.

Class Label	QGC-CNN (proposed)			Xception			ResNet50			EfficientNetB0		
	P	R	F1	P	R	F1	P	R	F1	P	R	F1
Glioma	0.98	0.9742	0.98	0.99	0.9892	0.99	0.99	0.9857	0.99	0.97	0.7821	0.87
Meningioma	0.98	0.9172	0.95	0.98	0.9692	0.98	0.99	0.9507	0.97	0.98	0.7235	0.83
No Tumor	0.99	0.9878	0.99	0.99	0.9892	0.99	0.99	0.9914	0.99	0.61	0.9875	0.76
Pituitary	0.93	0.9971	0.96	0.98	0.9995	0.99	0.97	0.9990	0.98	0.93	0.7885	0.86
**Macro Avg**	**0.97**	**0.97**	**0.97**	**0.99**	**0.99**	**0.99**	**0.98**	**0.98**	**0.98**	**0.87**	**0.82**	**0.83**

**P**, Precision; **R**, Recall; **F1**, F1-score. Bold values indicate the best-performing result for the corresponding metric.

Some important observations can be made at this level (from Table 7): Decisiveness in Healthy Tissue: The QGC-CNN model achieved a Precision of 0.99 for the “No Tumor” class, which is comparable to other heavier models. This confirms the effectiveness of the query-guided attention mechanism for filtering out the healthy anatomical tissues. Sensitivity to Pituitary Tumors: The model was able to obtain a Recall of almost 1.00 for the “Pituitary Tumors” class. This is important for the detection of pituitary tumors because the model should not miss any class in the screening process. Resilience to Class Imbalance: The QGC-CNN model was still able to obtain a high Precision of 0.98 for the “Meningioma” class, which is inherently difficult to classify due to the similarity of the features. In contrast, the “No Tumor” class of the EfficientNetB0 model had a low Precision of 0.61. This confirms the effectiveness of the cross-attention layer in the QGC-CNN for feature disentanglement. Each of the individual models demonstrate strong performance. However, integrating the models through different ensemble strategies can further enhance robustness and reliability.

### 4.4 Comparative study of ensemble paradigms

The performance of all ensemble methods was assessed using a five-fold stratified cross-validation scheme (Stone, 1974; Geisser, 1975; Kohavi, 1995; Hastie et al., 2009). All base models were trained using early stopping (patience = 5) and learning rate scheduling (ReduceLROnPlateau), which stabilized convergence and mitigated overfitting. However, training accuracy and training loss were not used for performance comparison, as they are optimistically biased. The OOF-based evaluation provides a more reliable estimate of real-world performance in medical image classification tasks. To provide an unbiased and leakage-free performance evaluation, all training performance metrics are calculated and reported solely based on the out-of-fold (OOF) predictions. The results of the OOF evaluation from training data of ensemble models are shown in Table 8. Although Bagging has the best Accuracy (0.9943) and Macro F1-score, the best **Stacking Ensemble** was chosen as the better model for the clinical application because of its much better probabilistic calibration.

**Table 8:** Out-of-fold (five-fold cross-validation) performance comparison of ensemble strategies.

Metric	Boosting	Bagging	Stacking (ours)
Accuracy	0.9859	0.9943	0.9878
Macro F1	0.9859	0.9943	0.9878
Macro recall	0.9859	0.9943	0.9878
Macro Spec.	0.9953	0.9981	0.9959
ROC-AUC	0.9990	0.9998	0.9994
MCC	0.9812	0.9924	0.9838
Kappa	0.9812	0.9924	0.9838
Log loss	0.0558	0.2819	0.0434

#### 4.4.1 Results overview

Table 8 presents the OOF performance comparison among the three ensemble strategies applied to the same set of base learners (Xception, EfficientNetB0, ResNet50, and QGC-CNN).

One of the most important drawbacks of the Bagging model is its high Log Loss of 0.2819. This shows that although Bagging is very accurate, it is not very well-calibrated and essentially becomes “over-confident” in its predictions. On the other hand, the Stacking model has a much lower Log Loss of 0.0434, demonstrating superior probabilistic calibration. Compared to the strongest standalone model (Xception), stacking reduced Log Loss by 17%, indicating improved confidence reliability without sacrificing predictive performance. This shows that the LightGBM meta-model is able to correctly detect the biases of the individual models, which is a very important requirement for any medical decision-support system, and is able to provide more “trustworthy” probability predictions. As shown in Table 9, the class-wise OOF performance comparison further demonstrates the effectiveness of the ensemble strategies across individual tumor classes.

**Table 9:** Comparative per-class OOF performance across ensemble strategies.

Class label	Precision	Recall	F1-score	Specificity
Boosting				
Glioma	0.99	0.9864	0.99	0.9976
Meningioma	0.98	0.9721	0.98	0.9940
No Tumor	0.99	0.9900	0.99	0.9971
Pituitary	0.98	0.9950	0.99	0.9924
Bagging				
Glioma	0.9971	0.9950	0.9961	0.9990
Meningioma	0.9900	0.9914	0.9907	0.9967
No Tumor	0.9964	0.9957	0.9961	0.9988
Pituitary	0.9936	0.9950	0.9943	0.9979
Stacking (Ours)				
Glioma	0.99	0.9900	0.99	0.9969
Meningioma	0.98	0.9800	0.98	0.9930
No Tumor	0.99	0.9900	0.9928	0.9964
Pituitary	0.99	0.9900	0.9885	0.9973

Analyses of the results indicates particular architectural vulnerabilities of the competing methods:

- Boosting performance gap: Although Boosting had a competitive Accuracy of 0.9859, it had the lowest Macro Specificity of 0.9953 among the three ensemble methods. This indicates that the sequential reweighting process of Boosting is slightly more susceptible to False Positives than the meta-learning approach.
- Bagging calibration risk: The first demerit of Bagging is its failure to account for base-learner bias. By applying a straightforward averaging rule, Bagging maintains the deep CNNs' over-confidence, resulting in the high Log Loss value reflected in the logs.

Among the tested methods, **Bagging** showed the best macro F1-score and ROC-AUC values, but the principle of aggregating results in **stacking's meta-learning** was found to be the most effective in reducing the False Positive Rate (FPR) while keeping the MCC at 0.9838. This shows that intelligent weighting of architectural diversity is better than just variance reduction for clinical safety.

#### 4.4.2 Testing result

To avoid overfitting during the last stage of model consolidation, the average convergence point from five-fold cross-validation is used to determine the number of epochs for each base architecture. The stack ensemble was then trained using the whole training dataset (*N* = 5, 600). While models that converge more quickly, like Xception, are trained for a shorter amount of time (average 16 epochs), more complex models, like QGC-CNN, are well-trained (average 23 epochs). This was tested on a rigorously independent, held-out test dataset (*N* = 1, 600). To further improve the diagnostic robustness, Test-Time Augmentation (TTA) was used during testing, with 5 stochastic augmentations (rotations, flips, and zooms) per image (Wang et al., 2019). The final stack ensemble resulted in a high diagnostic accuracy of 95.50% with a Macro F1-score of 0.9538. As evident from Table 10, the system shows good reliability performance on intricate statistical measures, with a Matthews Correlation Coefficient (MCC) (Matthews, 1975) of 0.9418 and a Cohen's Kappa of 0.9400, reflecting a competitive performance agreement between the predictions and the actual pathological results.

**Table 10:** Final stack ensemble evaluation summary (held-out test set).

Metric	Final ensemble value
Overall accuracy	0.9550
Macro F1-score	0.9538
Cohen's Kappa	0.9400
MCC	0.9418
Log loss	0.6067
Macro ROC-AUC	0.9904
Macro specificity	0.9850

A key performance criterion for a clinical brain tumor diagnosis system is high specificity to avoid a high False Positive Rate (FPR). The final system resulted in a Macro Specificity of 0.9851, with a specificity of 1.0 for Glioma and 0.9975 for Pituitary tumors. This indicates strong discriminative capability on this evaluated dataset with no false positives for these specific tumor types.

**Figure 4:**
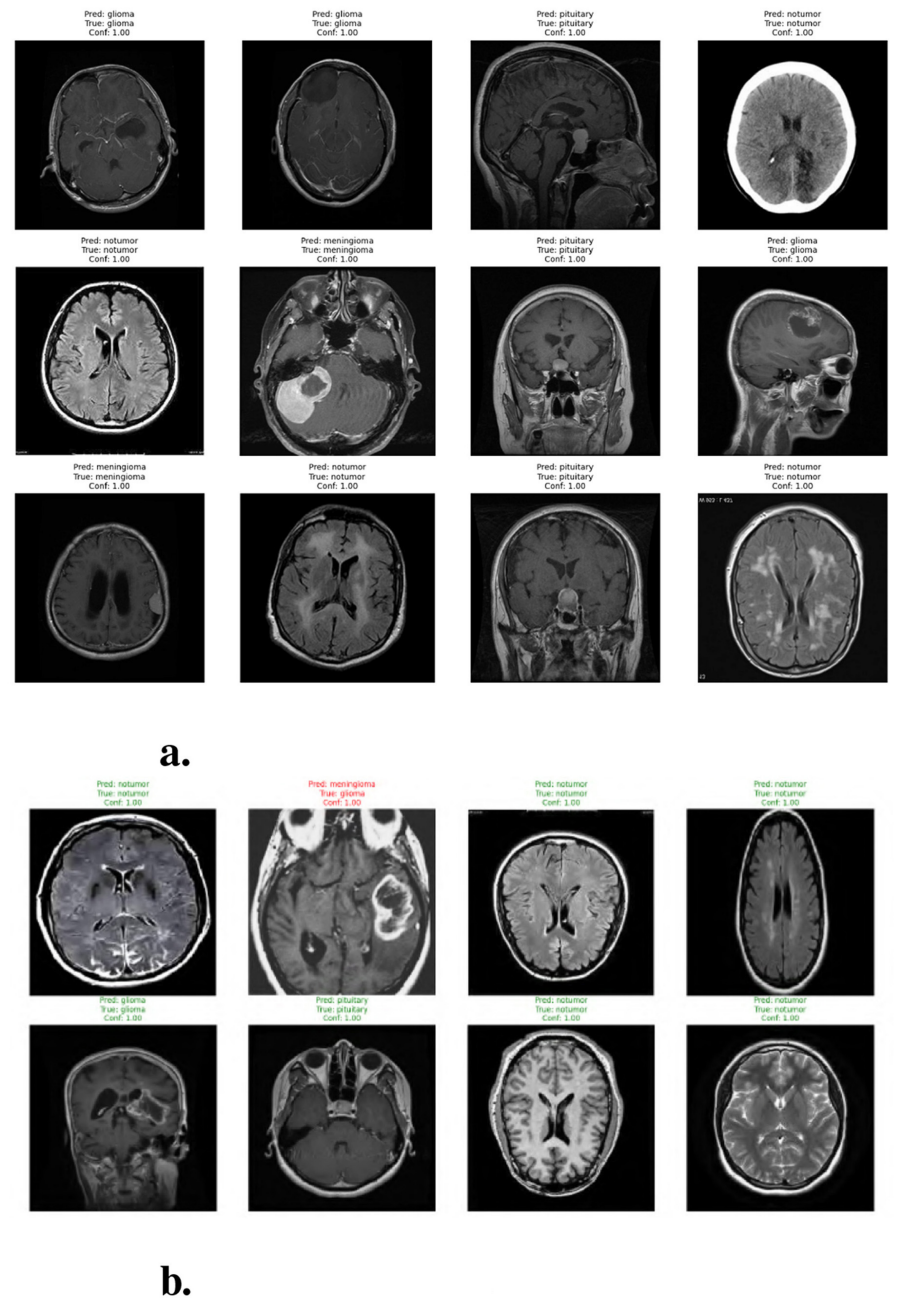
Representative sample predictions on the held-out test set. The model consistently achieves maximum confidence (1.00) for both tumorous and non-tumorous cases, demonstrating high probabilistic certainty. **(a)** Prediction Set 1. **(b)** Prediction Set 2.

As shown in Table 11, the confusion matrix shows that the model has 100% recall for healthy tissue (No Tumor), meaning that the model correctly identified all healthy images in the test data. Although there was some confusion between Glioma and Meningioma (38 cases), the model's 1.00 Precision for Glioma means that all predictions of Glioma made by the system achieved perfect precision on this dataset. The qualitative performance of the final stacked ensemble is demonstrated through example predictions on the held-out test set. As seen in the inference example in Figure 4, the model is highly confident (*Conf*:1.00) for diverse anatomical slices and tumor types.

**Table 11:** Class-wise performance breakdown on final test set.

Tumor class	Precision	Recall	F1-score	Specificity
Glioma	1.00	0.82	0.90	1.000
Meningioma	0.91	0.99	0.95	0.9642
No Tumor	0.93	1.00	0.96	0.9783
Pituitary	0.99	0.99	0.99	0.9975

**Figure 5:**
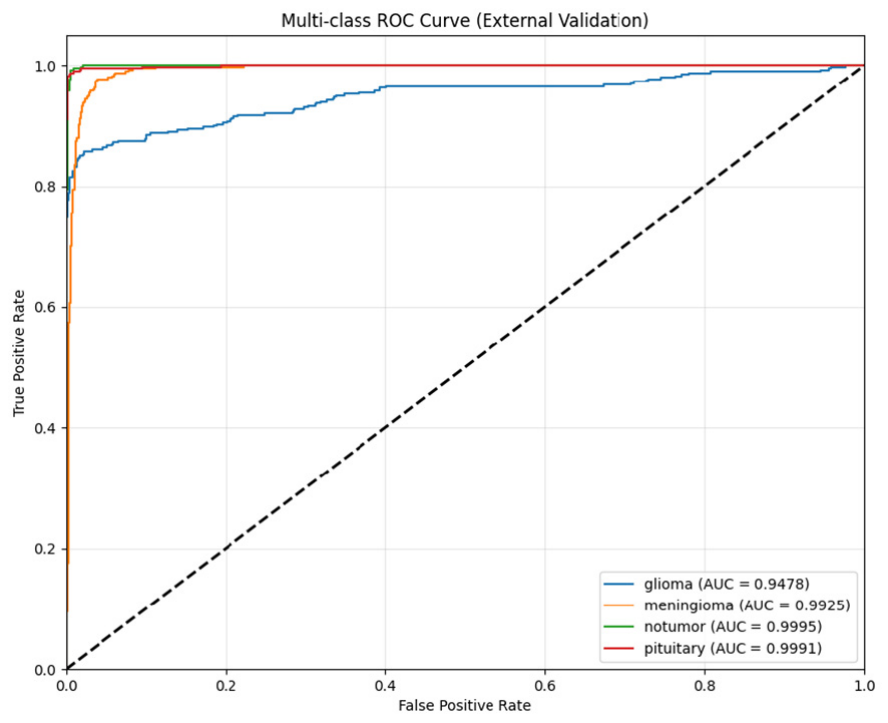
Multi-class ROC Curves for Held-Out Test Set. The competitive AUC values for “No Tumor” (0.9995) and “Pituitary” (0.9991) highlight the ensemble's superior discriminative performance.

Noticeably, the model retains perfect accuracy for the classification of “No Tumor” (healthy) brain tissue and Pituitary tumors in these examples. Although one example contains a misclassification between Glioma and Meningioma, it must be noted that the model did correctly classify the presence of a tumor, which is a highly important aspect for secondary clinical analysis. The discriminative power of the ensemble was further confirmed using Multi-class Receiver Operating Characteristic (ROC) curves. The Area Under the Curve (AUC) for each class gives a quantitative estimate of the model's discriminative power to distinguish between a particular tumor type and all other classes. As shown in Figure 5, the system has recorded high AUC values: No Tumor: 0.9995, Pituitary: 0.9991, Meningioma: 0.9925, Glioma: 0.9478. The close-to-ceiling AUC value for the “No Tumor” class (0.9995) is particularly important in the context of clinical screening, as it further confirms the system's nearly perfect power to exclude malignancy in healthy patients with almost no false alarms. Even in the most difficult tumor class (Glioma), the AUC value of 0.9478 is a strong indicator of high diagnostic accuracy. Beyond quantitative performance, it is important to analyze the internal decision-making behavior of the model.

### 4.5 Qualitative evaluation of feature representations and interpretability

**Figure 6:**
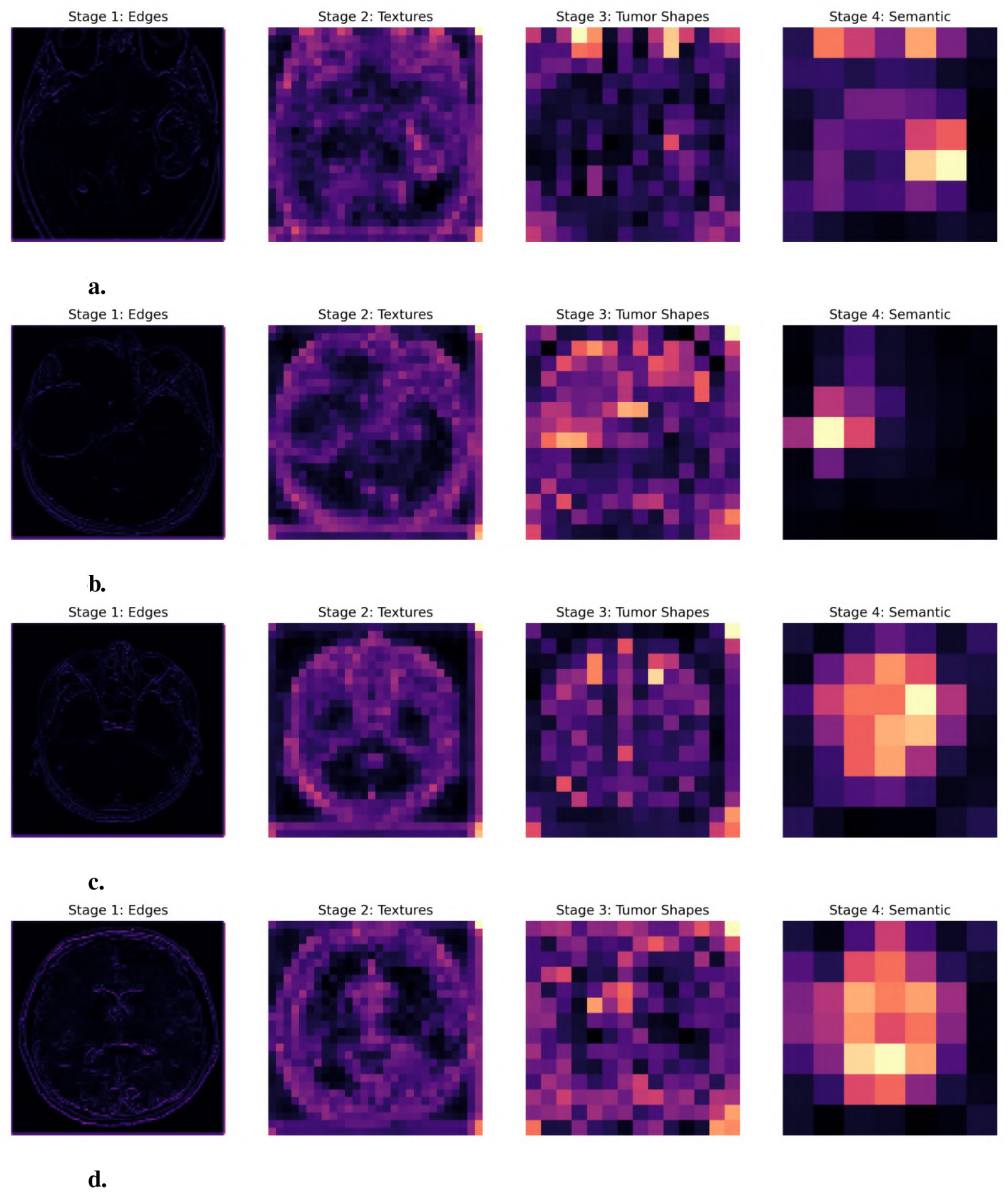
Hierarchical feature extraction across four diagnostic classes. The progression from Stage 1 to Stage 4 demonstrates the model's ability to transition from low-level edges to class-specific pathological biomarkers while successfully ignoring non-pathological anatomical noise. **(a)** Glioma: Transition to diffuse infiltrative pattern isolation. **(b)** Meningioma: Resolution of well-defined globular mass. **(c)** Pituitary: Activation at the cranial base. **(d)** No Tumor (Con trol): Diffused structural symmetry.

To further support the quantitative results, the qualitative results are analyzed to gain more insights into the learning of the internal feature of the proposed architecture. The observations from the qualitative results of the proposed architecture are as follows:

1. **Baseline CNN localization behavior:** Grad-CAM visualizations showed that the baseline CNN models generally localized the tumor regions well. However, the attention was not always crisp but spread out beyond the tumor regions for some of the classes. Activation analysis of the stage-wise models showed the progression from low-level edge features to texture features and finally to the regions of interest inside the tumor. Feature encoding sparsity was found to be low for all the classes in the semantic layers.
2. **Query-guided attention patterns:** Query tokens showed high attention values localized inside the regions of interest for the tumor. However, the maximum attention values for the query tokens were not the same for all the classes. Concentration values showed that the attention was not uniform for the query tokens. This confirms the hypothesis that the cross-attention is a region-selective aggregation mechanism.
3. **Latent space separability:** t-SNE plots (van der Maaten and Hinton, 2008) showed visually separable clusters for the tumor classes. Quantitative analysis showed the following: Competitive silhouette scores (Rousseeuw, 1987) for the models. Lower intra-class variance for some of the architectures. Higher inter-class centroid distance for some of the architectures.
4. **Comparative learning efficiency:** Mid-stage activation overlays indicate that different architectures resolve tumor structures with varying spatial focus. The QGC-CNN model demonstrates consistent region-focused activation aligned with tumor morphology. This list of qualitative analysis is discussed in this sub section. The observations provided an intuitive understanding of how different architectures learn and represent tumor-specific features. Initially, to illustrate the hierarchical feature learning behavior of our QGC-CNN model across different tumor classes is presented in Figure 6. The Figure 6 presents the stage-wise activation patterns.

As seen in Figures 6a–d, there is an understandable hierarchy in abstraction. At initial stages (Stage 1 and Stage 2), model has its concentration on universal low-level features like edges in cortex and bone tissues. At Stage 3 (Tumor Shapes) and Stage 4 (Semantic), the model shows high fidelity in class-specific discernment. When considering the tumor-positive datasets (Glioma, Meningioma, and Pituitary), the activations localize well, effectively ignoring the healthy parenchyma. Interestingly, in the control class “No Tumor,” as depicted in Figure 6d, the Stage 4 representation shows symmetrical and normal structure without any abnormal high-intensity clusters. This essentially shows the QGC-CNN's ability to focus on tumor-specific information rather than detecting abnormalities in the brain. Although Figure 6a (Glioma) displays the infiltrating pattern along the border of tissues, Figure 6b (Meningioma) reveals a sharper as well as more globular pattern. Such uniqueness are reinforced in the Transformer Attention Maps. The “learnable query tokens” of the model “actively interrogate” all areas of the tumor in all pathological tissues (Tokens 4 and 8), but fail to do so in the control tissues, as presented in Figure 6d. This behavior suggests that the attention mechanism selectively focuses on diagnostically relevant regions rather than uniformly scanning the entire image.

**Figure 7:**
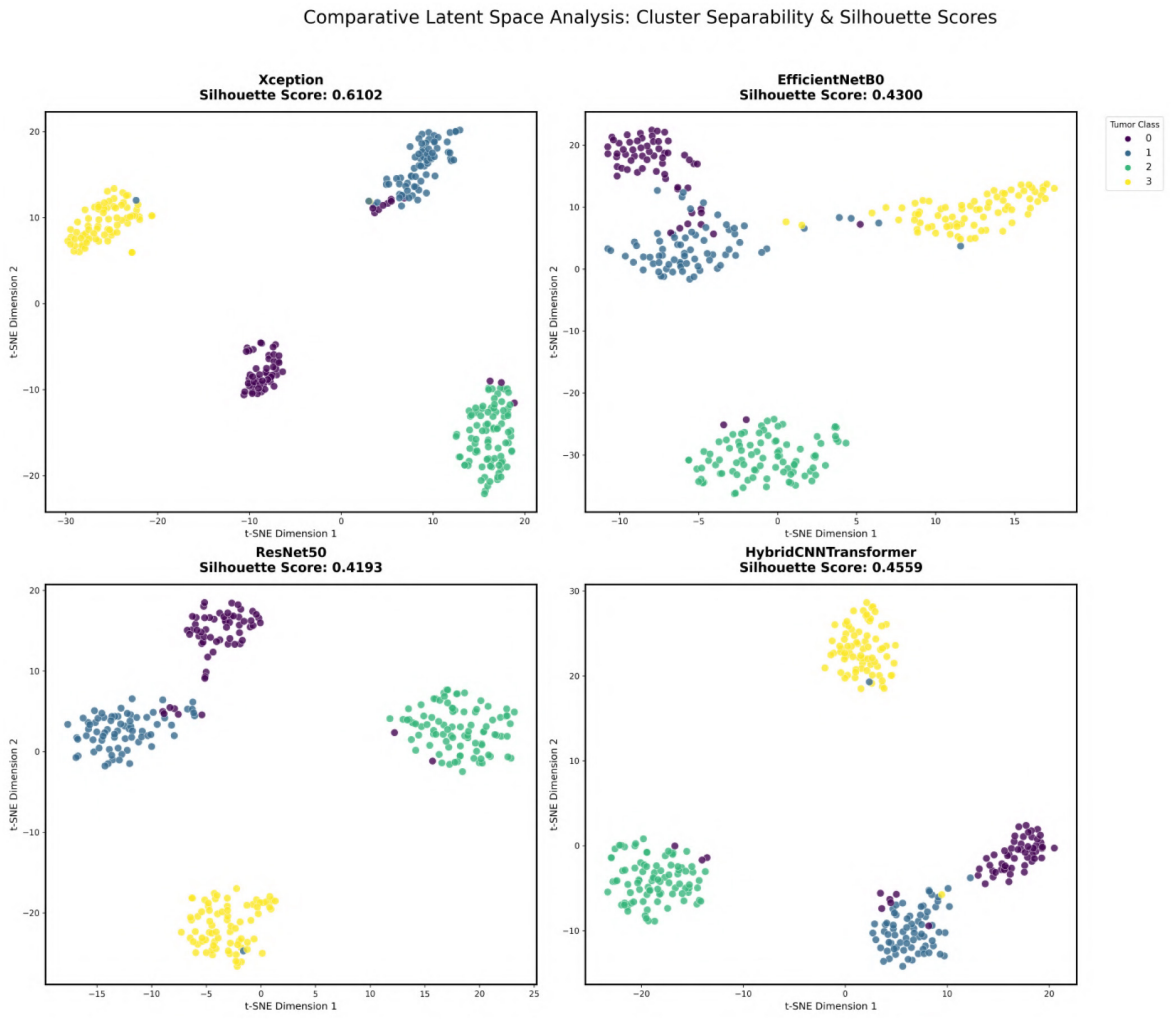
t-SNE visualization of the learned representations of the 512-dimensional features. The spatial separation of each class indicates the model's capacity to extract pathological features that are very distinct and can be linearly separated.

While the above analysis provides spatial and visual evidence of the model's feature learning behavior, it is equally important to evaluate how well these learned representations are organized in the feature space. The quality of such learned features is further ascertained by t-SNE (t-distributed Stochastic Neighbor Embedding) (van der Maaten and Hinton, 2008) analysis of the penultimate layer. As seen in Figure 7, the QGC-CNN displays a significantly better Inter-Class Distance of 28.99, which is substantially higher than the baseline models such as ResNet50 (13.92). This large inter-class variance, with very little overlap between histologically similar tumor classes, provides qualitative evidence of improved separability.

Further, these findings have been reinforced by quantitative latent space metrics provided in Table 12 that illustrate the superiority of the proposed model in inter-class separation with controlled intra-class variance.

**Table 12:** Comparative latent space metrics: cluster quality and separability.

Metric	Xception	EfficientNetB0	ResNet50	QGC-CNN
Silhouette score	0.6102	0.4300	0.4193	**0.4559**
Intra-class variance	0.2163	0.3712	0.3905	**0.3352**
Inter-class distance	15.97	14.78	13.92	**28.99**

Bold values indicate the best-performing result for the corresponding metric.

Besides separability, the task-aligned behavior of the proposed model is also tested using sparsity and attention concentration metrics. As depicted in Table 13, Glioma and Pituitary classes show near-zero sparsity in the last stage, which demonstrates strong alignment with pathological features and suppression of irrelevant anatomical information.

**Table 13:** Sparsity and attention concentration metrics for QGC-CNN semantic features.

Class	Final stage sparsity	Max attention focus	Mean token conc.
No Tumor	0.000064	0.3589	0.9660
Glioma	0.000000	0.1809	0.9633
Pituitary	0.000000	0.1459	0.9061
Meningioma	0.000064	0.1680	0.9857

The “Task-Aligned” characteristic of our model is measured in terms of Sparsity metrics at the semantic level. Glioma and Pituitary obtained the Final Stage Sparsity as 0.0000, signifying complete alignment with pathological biomarkers by suppressing anatomical noise. This is further emphasized in the Transformer Attention Maps, where the “learnable query tokens” (namely, Tokens 4 and 8) specifically probe the boundaries and nuclei of the lesions. In the class category “No Tumor,” the tokens resort to a general anatomical sweep, ensuring structural symmetry and a non-zero sparsity measure of 0.000064, thus validating the model's capacity to distinguish between pathological and healthy conditions.

**Figure 8:**
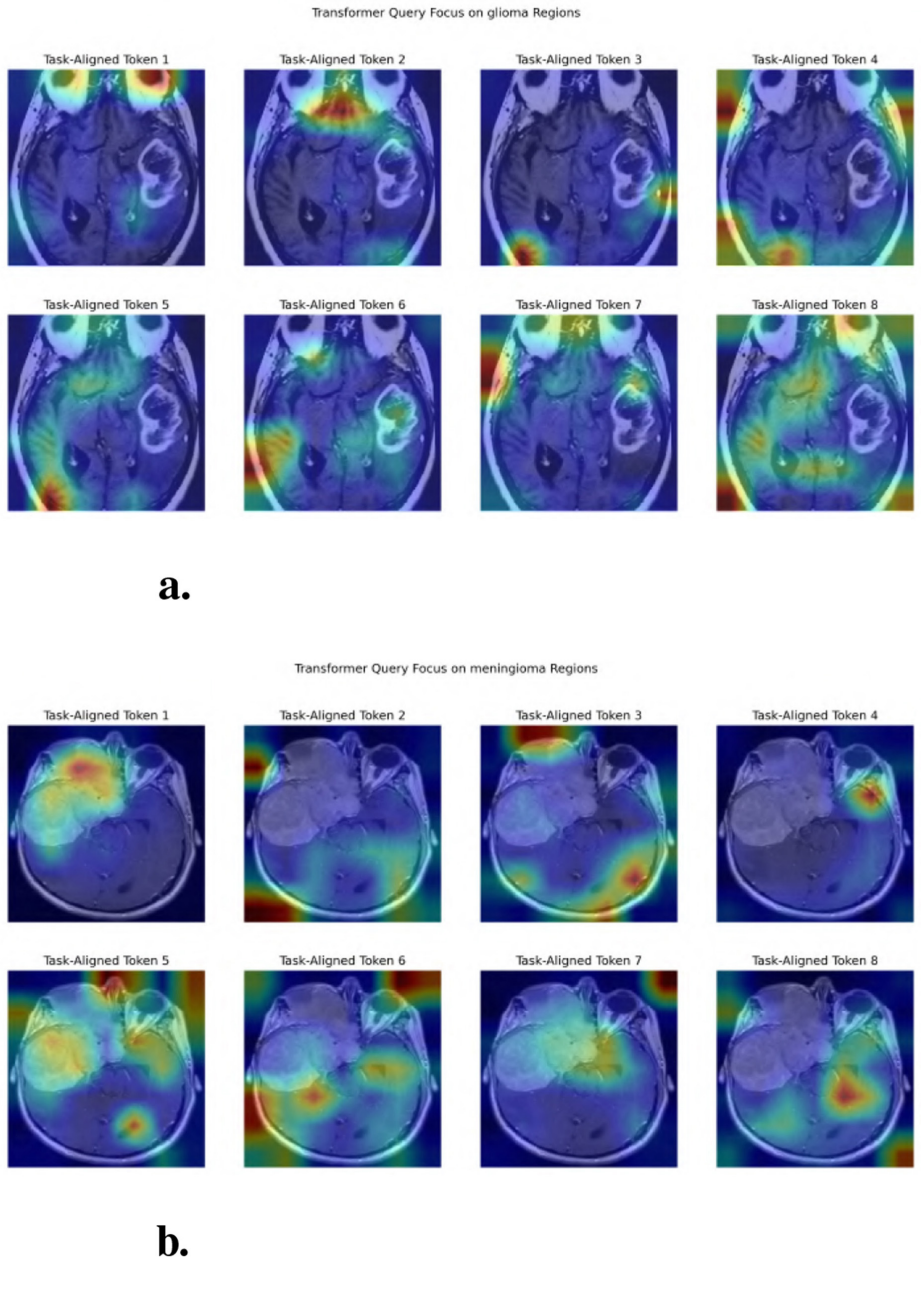
Hierarchical feature extraction across diagnostic classes. The progression from Stage 1 to Stage 4 demonstrates the model's ability to transition from low-level edges to class-specific pathological biomarkers while suppressing non-pathological anatomical noise. **(a)** Glioma: Transition to diffuse infiltrative pattern isolation. **(b)** Meningioma: Resolution of well-defined globular mass.

Building upon the previous qualitative and latent space analysis, the task-aligned behavior of the proposed QGC-CNN architecture is further investigated through cross-attention visualization. An observation is made from attention diagrams that tokens do not have redundant behavior rather they specialized in different subtasks. For example, in the samples for Meningioma (Figure 8b) and Glioma (Figure 8a), Token 4 and Token 8 have high-intensity concentration on lesion boundary and core, respectively.

**Figure 9:**
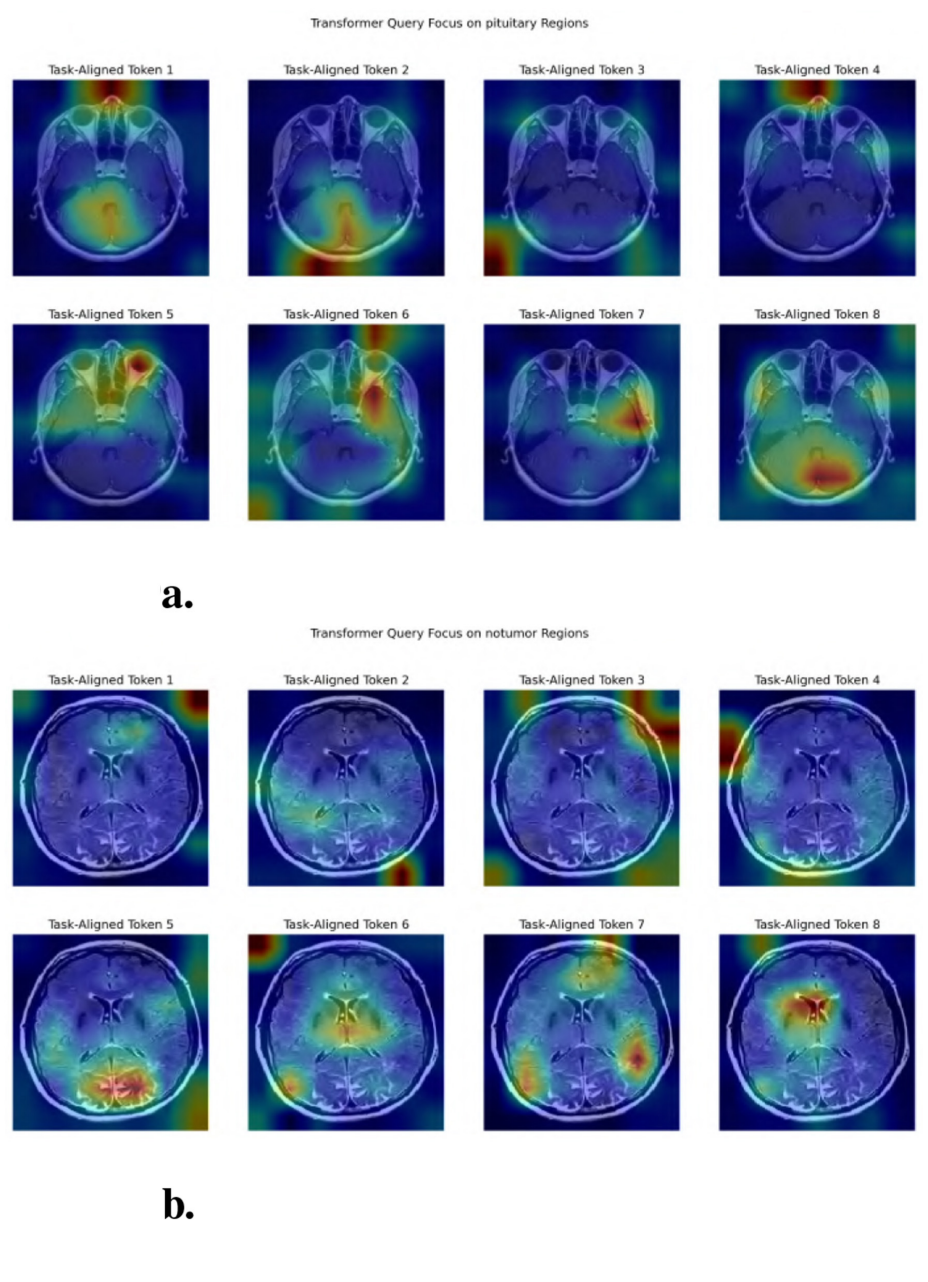
Hierarchical feature extraction across four diagnostic classes. The progression from Stage 1 to Stage 4 demonstrates the model's ability to transition from low-level edges to class-specific pathological biomarkers while successfully ignoring non-pathological anatomical noise. **(a)** Pituitary: activation at the cranial base. **(b)** No Tumor (Control): Diffused structural symmetry.

These visualizations provide insight into how different query tokens specialize in capturing distinct tumor characteristics across classes. For the Pituitary class (Figure 9a), the query tokens show a high intensity focus on the cranial base. This is clinically significant as it corresponds to the anatomical region where pituitary adenomas arise, thus confirming that the “learnable queries” have successfully learned to focus on class-specific landmarks. In the “No Tumor” control set (Figure 9b), the tokens display a fallback strategy, where they go back to the general anatomical scan if no lesion is found. This exploratory pattern adds a degree of clinical interpretability to the architecture, which enables the physician to check if the model is indeed “searching” for biomarkers instead of simply scanning pixels. On the whole, these qualitative analyzes further verify that the proposed QGC-CNN approach is able to learn structured and clinically meaningful feature representations effectively, consistent with the strong quantitative results. While the above analysis highlights the effectiveness of the query-guided attention mechanism in the proposed architecture, it is equally important to compare this behavior with conventional CNN-based localization techniques.

#### 4.5.1 Grad-CAM visualizations

**Figure 10:**
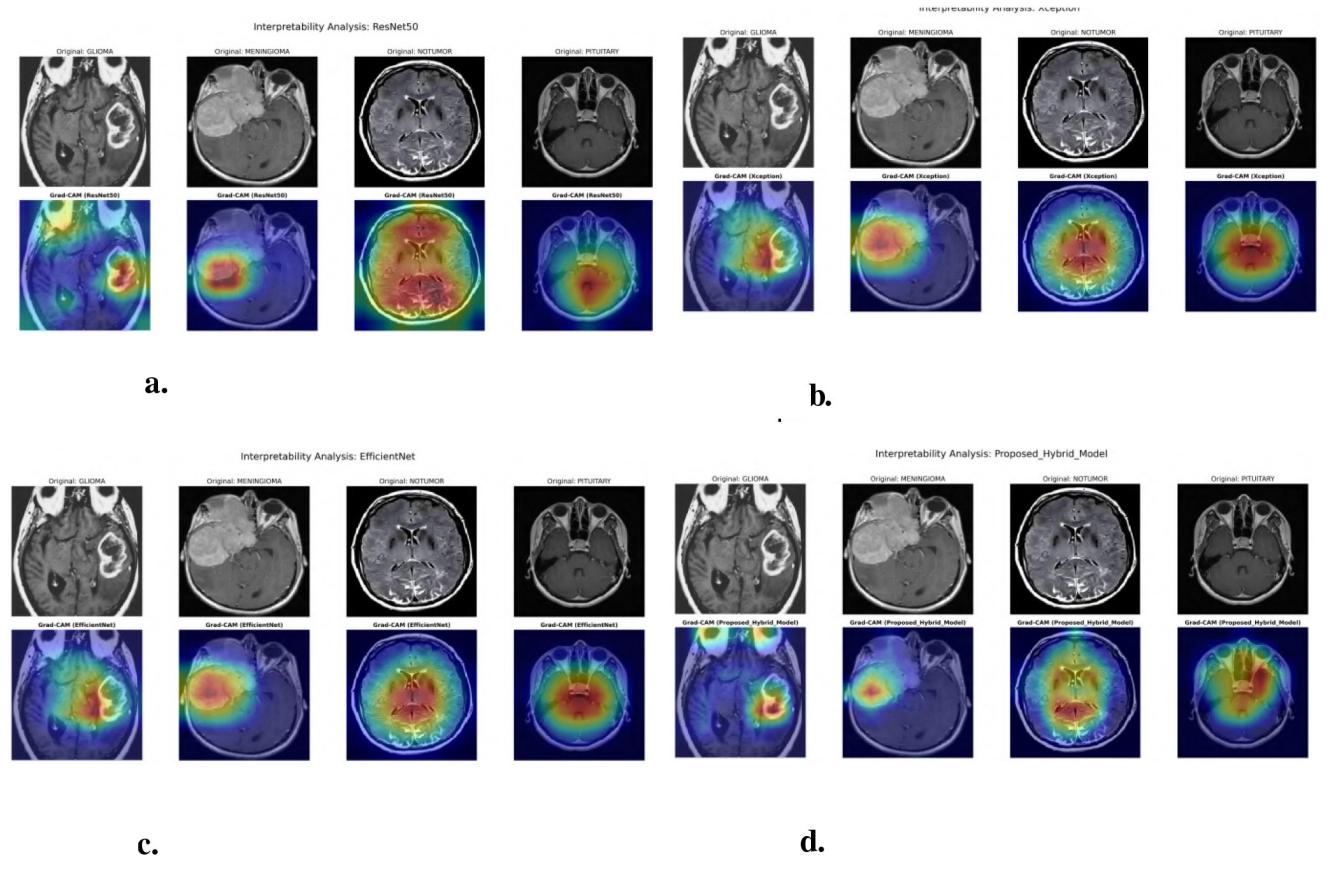
Grad-CAM visualizations for each class emphasizing the parts of the MRI images that contributed the most to the model's predictions, from left to right: glioma, meningioma, no tumor and pituitary. **(a)** Grad-CAM visualizations comparing feature localization in Resnet. **(b)** Grad-CAM visualizations comparing feature localization in Xception. **(c)** Grad-CAM visualizations comparing feature localization in EfficientNet. **(d)** Grad-CAM visualizations comparing feature localization in QGC-CNN.

The Grad-CAM visualizations for baseline models and the proposed QGC-CNN are presented in Figure 10. The standard convolutional networks (Xception, ResNet50, and EfficientNetB0) follow a passive activation pattern. Although they are able to correctly point out the general areas of interest,the Grad-CAM maps may not have a precise anatomical correspondence: Xception & ResNet50: These networks tend to produce general areas of activation that tend to include healthy brain areas, thus suggesting a global texture-based approach rather than a morphology-based approach. EfficientNetB0: Although it tends to have a better focus on lesions, it still preserves considerable activation noise in the “No Tumor” control group, thus suggesting a potential risk of false-positive triggers.

The validation through the literature was carried out using the comparison of the spatial saliency heatmaps of Grad-CAM with the pathological landmarks mentioned in the WHO Classification of Tumors of the Central Nervous System from 2021 (Louis et al., 2021, 2016). This approach aligns with prior studies in explainable medical AI, where visual explanations are evaluated based on their correspondence with clinically relevant anatomical regions (Tjoa and Guan, 2020; Arun et al., 2021). For neuro-oncology workflows, Grad-CAM supports model auditing, error detection, and clinician-AI collaboration, especially in tumor classification and radiogenomic studies (Lundberg et al., 2020). As shown in Figure 10, Row (d), the QGC-CNN exhibits higher spatial accuracy when compared to the baseline neural networks. The rationale behind the localization of the model correlates well with the clinical practice for the following reasons: **Glioma:** The high-grade gliomas are typically diagnosed clinically based on heterogeneous contrast enhancement and peritumoral edema (Louis et al., 2021; Kickingereder et al., 2016; Law et al., 2003). In case of the baseline networks (Rows a–c), there is a diffuse activation in the healthy hemisphere. On the contrary, the QGC-CNN localizes around the tumor margins. This suggests that the model has managed to learn the most malignant regions of the lesion, rather than the whole image texture. **Meningioma:** Meningiomas are usually well-defined tumors, attached to the dura (Goldbrunner et al., 2016).

In Grad-CAM visualization for meningioma, the proposed network highlights only the tumor with all attachment points, unlike EfficientNetB0 that visualizes the adjacent cortical noise.

**Pituitary:** It is imperative that pituitary macroadenomas be localized at the bottom of the brain. The QGC-CNN focuses on the sella turcica area only, highlighting high sensitivity toward crucial anatomical markers (Molitch, 2017).

From a clinical perspective, Grad-CAM visualizations are not intended to replace expert judgment but to serve as an assistive verification mechanism. In practice, clinicians could utilize such heat maps to verify whether the predictions made by the model agree with anatomical and pathological facts (Tjoa and Guan, 2020). The inconsistency between the heat map and lesion area suggests that the network may make incorrect predictions. On the other hand, a high level of consistency provides more confidence about the predictions made by the network. In addition, it is evident that visualization techniques significantly increase the reliability of clinical artificial intelligence models when incorporated into the system as the second tier of validation. In this case, the proposed QGC-CNN is considered a clinically-assistive framework, where visual explanations provide additional assurance for decision-making. In addition to evidence from the literature, clinical evidence was sought by consulting an experienced physician. Based on the expert's opinion, it appears that the visual guidance provided by the heatmap is intuitively related to the anatomy of the organs and can help clinicians verify that the model prediction is consistent with the radiologically relevant area, not just some meaningless artifacts. The expert also pointed out that the interpretation of models using such visual guidance could be especially helpful in early diagnosis and primary healthcare practices, where clinicians who are not experts in this field need support for identifying suspicious signs to refer patients to specialists. It should be noted, however, that the above results are still not a clinical study but rather an expert evaluation. In the future, the focus will shift to structured testing by radiologists and multi-expert consensus. On the whole, both qualitative and quantitative analysis of the proposed QGC-CNN model suggest that it is efficient in learning discriminative and clinically relevant features. In order to assess the robustness and generalization potential of the proposed model, its performance is evaluated on external dataset in the next section.

### 4.6 External validation

In order to assess the generalizability of the proposed approach on a wider range beyond the specific dataset, the external validation process was conducted on the brain tumor MRI dataset available on Mendeley Data (Hira et al., 2026). The dataset includes T1-weighted contrast-enhanced MRI images. No fine-tuning was performed on the external dataset, allowing evaluation on an independent test distribution.

#### 4.6.1 Dataset description

The external dataset includes ~11,148 pre-processed T1-weighted contrast-enhanced MRI images that belong to one of the following four categories: Glioma, Meningioma, Pituitary tumor, and No tumor. It should be noted that unlike the initial Kaggle dataset, the external dataset demonstrates heterogeneity of real-world data because of varying image acquisition parameters. Authors of this dataset, split the data into 80% training data and 20% testing data. Images are organized into subfolders by class. A total number of 1,857 images (only testing data) was employed for analysis. The breakdown of each class is presented as follows: **Glioma: 603 images; Meningioma: 436 images; Pituitary tumor: 429 images; No tumor: 389 images**. All images were resized to 224 × 224 and standardized similarly as per the training procedure. No data augmentation technique was used for external validation to maintain neutrality in the process. The entire stack of the models that had been trained previously was tested on the external dataset without any further tuning. This stringent process is carried out in order to ascertain the generalization ability of the models. The novel approach demonstrate strong performance on the external dataset; the obtained results are as follows:

- Accuracy: 93.86%.
- Macro F1-score: 0.9407.

The detailed class-wise performance is presented in Table 14.

**Table 14:** External validation performance on mendeley dataset.

Class	Precision	Recall	F1-score
Glioma	0.99	0.84	0.91
Meningioma	0.89	0.96	0.92
No Tumor	0.89	1.00	0.94
Pituitary	0.98	1.00	0.99

**Figure 11:**
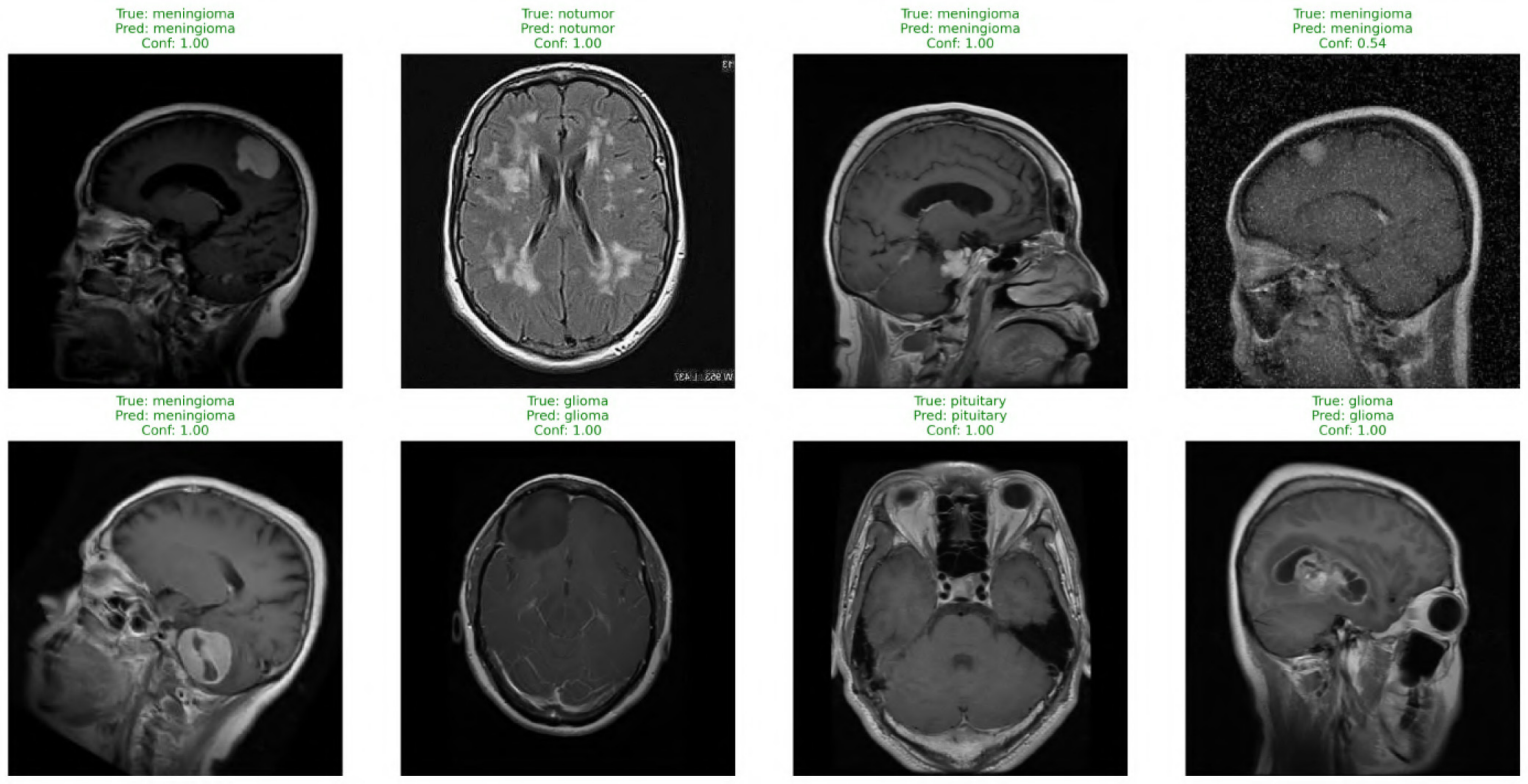
Sample predictions on the external Mendeley dataset showing true labels, predicted labels, and confidence scores.

These results show that despite the domain shift, the model is still able to classify well. Of particular importance, recall for “No Tumor” and “Pituitary” classes equals one, meaning that the probability of misclassification of those classes will be minimal in the clinical diagnosis process. Recall for “Glioma” class is reduced (0.84) because of the higher inter-class variance and difference in appearance between tumors in multiple institutions, making domain shift challenging. The external validation results suggest that the QGC-CNN based ensemble developed in this study is not overfitted and has generalizability on different datasets. A reduction in performance metrics by approximately 4% can be expected when considering a domain shift problem. These results directly solve the concerns raised in the review about dependency on one dataset only. Future research might focus on further increasing performance metrics on an independent dataset using methods such as domain adaptation or federated learning techniques. To complement the quantitative evaluation, qualitative analysis was performed on representative samples from the external Mendeley dataset. Figure 11 presents example predictions of the proposed model across different tumor classes.

The model demonstrates strong visual consistency in correctly identifying diverse tumor types, including Glioma, Meningioma, and Pituitary tumors, as well as healthy (No Tumor) cases. Most predictions are associated with high confidence scores (close to 1.00), indicating strong model certainty even under cross-dataset conditions. Notably, the model successfully captures:

- Distinct tumor boundaries in glioma cases.
- Well-defined extra-axial masses in meningioma.
- Characteristic pituitary region abnormalities.

A problematic instance was observed when predicting a meningioma case with less accuracy (0.54). This implies that there was some level of confusion brought about by the noise or abnormality in the images. It clearly indicates the challenges that come with the process of making generalization on varied datasets. The observed difference in accuracy between the two groups by ~1.6% is directly attributed to the “Domain Shift” phenomenon associated with multi-center data. The Mendeley dataset has added noise in terms of sensors used and T1-weighted contrast enhancement timings which deviate from the Kaggle dataset. The particular drop in Glioma recall of 0.84 indicates that despite the robustness of the QGC-CNN framework, minute differences could be due to slice thickness or acquisition settings across different centers continue to present a barrier for flawless implementation.

The above differences show how the performance of the model can be affected by domain shift and imaging variability. Although the proposed QGC-CNN architecture shows resilience to these challenges, this analysis highlights the significance of testing models under different acquisition scenarios for better generalization.

In order to test the performance of the proposed method against other advanced models, a detailed comparison is made in the next section.

### 4.7 Comparison with hybrid architectures

To evaluate the performance of the proposed QGC-CNN architecture, a comprehensive comparison was conducted against state-of-the-art and recently developed deep learning models. All models were trained using an equal setting for the experiment: the same dataset, same preprocessing procedure, same size of input (224 × 224), and five-fold cross validation.

1. **Baseline CNN architectures** Three widely adopted convolutional neural networks were selected as baseline models:
  - **ResNet50:** A residual learning-based architecture that enables deeper network training through identity skip connections.
  - **Xception:** A depthwise separable convolution-based model known for its efficiency and strong feature extraction capability.
  - **EfficientNetB0:** A lightweight architecture that scales depth, width, and resolution using compound scaling.
  These models represent strong CNN baselines commonly used in medical image classification tasks.
2. **Transformer and hybrid architectures** To provide a more critical comparison with recent trends, transformer-based and hybrid models were also implemented:
  - **ConvNeXt:** A modern CNN inspired by the Vision Transformer approach, with features such as big kernels and layer normalization included (Barber et al., 1996).
  - **TransUNet-like hybrid:** The CNN-Transformer hybrid model that uses ResNet50 to extract the features and then reshape them into tokens and pass them through the Transformer encoder blocks. In this way, it is possible to capture not only the local but also the global information (Chen et al., 2021).
  - **CNN-ViT hybrid:** Deeper CNN-Transformer Hybrid model in which the convolutional feature maps are reduced to an embedding space and passed through multi-headed self-attention blocks. This helps capture the global properties while still benefiting from CNN inductive biases (Dosovitskiy et al., 2021).
3. **Proposed QGC-CNN model** The proposed QGC-CNN model advances the typical hybrid approaches by incorporating the query-guided attention approach, which concentrates on the highly discriminative parts of the MRI images. In contrast to the conventional self-attention approach, which assigns the same weights to all tokens, the query-guided tokens play a role in guiding the feature extraction process.

#### 4.7.1 Experimental consistency

To ensure fairness and reproducibility:

- All models were trained using exactly the same train-test split approach (5-fold stratified cross-validation).
- Only the training folds were augmented with respect to data augmentation.
- Learning strategies such as learning rate scheduling and early stopping were maintained the same across the models.

The models were evaluated using multiple performance metrics, including: Accuracy, Macro F1-score, Cohen's Kappa, Matthews Correlation Coefficient (MCC), Log Loss, ROC-AUC, and Specificity. This multi-metric evaluation ensures a comprehensive assessment of both classification performance and reliability, which is critical for clinical applications.

A comparison table for the proposed QGC-CNN model and several other cutting-edge models is presented in Table 15. It is analyzed that the performance of the proposed architecture proves to be remarkably good in terms of all metrics used. Of the baseline CNN models, Xception gave quite impressive results with excellent ROC-AUC and low log loss, thus demonstrating good calibration. Nevertheless, its accuracy and F1-score are still low. The Transformer-based models along with hybrid architectures like CNN-ViT, TransUNet, and ConvNeXt performed better, it is due to their capability to learn long-range dependencies. It was also observed that TransUNet and ConvNeXt performed very well. The accuracy of the QGC-CNN approach was 94.63%, whereas its macro F1-score stood at 0.945. The proposed model proved reliable by having excellent Kappa coefficient values (0.928) and MCC (0.929). It should also be noted that the high value of specificity obtained (0.982) plays a vital role in eliminating false negatives. The best performance was recorded through the application of QGC-CNN ensemble technique, which combines predictions made through cross-validation processes. This method recorded the best accuracy, 95.50%, and macro F1 score, 0.9538. It also outperformed other existing algorithms in terms of the above performance metrics. While there were impressive performances overall, a further analysis by class shows that glioma remains one of the difficult-to-classify classes due to overlapping visual features of tumors. On the other hand, the no tumor and pituitary classes performed exceptionally well in classification tasks. Overall, the findings suggest that the suggested query-guided attention model achieving competitive performance compared to traditional CNN and hybrid architectures, while the ensemble variant attains the highest overall performance. While the above results demonstrate strong classification performance, practical deployment also requires consideration of computational efficiency, which is analyzed in the following section.

**Table 15:** Performance comparison of proposed QGC-CNN with other models on held-out test set.

Model	Accuracy	Macro F1	Kappa	MCC	Log loss	ROC-AUC
EfficientNetB0	0.9337	0.9312	0.9117	0.9147	0.4871	0.9919
ResNet50	0.9400	0.9385	0.9200	0.9218	0.3895	0.9896
Xception	0.9450	0.9440	0.9267	0.9282	**0.2433**	**0.9928**
CNN-ViT Hybrid	0.9481	0.9471	0.9308	0.9320	0.3047	0.9902
TransUNet	0.9531	0.9525	0.9375	0.9388	0.3505	0.9920
ConvNeXt	0.9531	0.9525	0.9375	0.9388	0.3505	0.9920
**QGC-CNN (Proposed)**	0.9463	0.9450	0.9283	0.9299	0.2690	0.9924
**QGC-CNN Stack Ensemble**	**0.9550**	**0.9538**	**0.9400**	**0.9418**	0.6067	0.9904

Bold values indicate the best-performing result for the corresponding metric.

### 4.8 Computational complexity analysis

Besides performance assessment, a complexity analysis is performed to determine the efficiency of the proposed method. In the complexity analysis, the number of parameters, GFLOPS, and the runtime per batch size are considered. The values of accuracy are reported for the performance of the algorithm on the held-out test dataset for consistent comparison with other algorithms.

As seen from Table 16, it is evident that although conventional deep learning algorithms like ResNet50 and Xception can deliver high classification accuracy, their computational requirements are very high (7.73–9.11 GFLOPs). In addition, the hybrid models of CNNs and Transformers such as CNN+ViT and TransUNet, which have higher classification accuracy, suffer from higher computational complexity. However, in comparison, the proposed QGC-CNN algorithm can offer competitive classification accuracy (94.63%) with lower computational complexity (0.83 GFLOPs and 5.5M parameters). In other words, the QGC-CNN model offers a computational efficiency improvement of about 10 times over CNN and hybrid models, without much loss in accuracy. In addition, compared to transformer-based approaches like ConvNeXt and TransUNet, which are more accurate but have substantially higher computational requirements, the proposed model strikes a balance in terms of computational efficiency. It means that the use of the query-guided attention method allows for efficient feature extraction without a significant increase in complexity. The application of an ensemble method further enhances the results, producing the highest test accuracy score of 95.50%. As always, better results are accompanied by an increase in the computational cost (18.46 GFLOPs). So, the QGC-CNN can be recommended for use in resource-limited settings, while an ensemble approach would be preferred where computational costs are not an issue. However, the actual execution time can depend on particular factors, and so GFLOPs and parameters' number are deemed the key measures of computational cost.

**Table 16:** Comparison of computational complexity and performance.

Model	Params (M)	GFLOPs	Time (s/batch)	Test Accuracy (%)
*Single models*				
EfficientNetB0	5.3	0.79	0.1065	0.9337
ResNet50	23.85	7.73	0.1229	0.9400
Xception	21.12	9.11	0.1393	0.9450
CNN+ViT Hybrid	24.21	7.76	0.2925	0.9481
TransUNet	34.09	8.78	0.6498	0.953
ConvNextTiny	27.8	8.78	0.6848	0.953
**QGC-CNN (Ours)**	**5.50**	**0.83**	**0.1138**	**0.9463**
*Ensemble model*				
**QGC-Ensemble (Final)**	54.69	18.46	0.4831	**0.9550**

Bold values indicate the best-performing result for the corresponding metric.

### 4.9 Statistical analysis

A statistical significance test was performed using the test set to objectively measure the relative performance of various models. Different statistical significance tests have been performed assuming both parametric and non-parametric conditions. McNemar's test (McNemar, 1947), paired *t*-tests (Student, 1908), and Wilcoxon signed-rank tests (Wilcoxon, 1992) were applied to verify the results. Furthermore, confidence intervals based on the bootstrap method (Efron, 1992) were calculated to confirm their stability. The statistical tests were performed on paired prediction outputs of the models on the same test samples. From the results, it is evident that the stacking ensemble achieves statistically comparable performance to high-performing baseline models under the given evaluation setting, including models such as ResNet50, Xception, and the QGC-CNN. Though there are numerical differences in accuracy, the statistical analysis performed by McNemar's test, paired *t*-test, and Wilcoxon signed-rank test is not statistically significant. The Wilcoxon signed-rank test is reported for the primary comparison between the QGC-CNN and the stacking ensemble to validate consistency under non-parametric assumptions, while McNemar's and paired *t*-tests were applied across all model comparisons. The overlap in the 95% confidence intervals further supports the absence of statistically significant differences among top-performing models. On the other hand, the proposed ensemble method proves significantly better than the EfficientNetB0 model (*p* < 0.001). The detailed statistical comparison results between models are summarized in Table 17, highlighting their relative performance and significance levels.

**Table 17:** Comparison of model performance and statistical significance on the test set.

Model	Accuracy	Kappa	Comparison with ensemble	Significance
ResNet50	0.9932	0.99	Comparable	*p*>0.05
Xception	0.9935	0.99	Comparable	*p*>0.05
TransUNet	0.9830	0.98	Comparable	*p*>0.05
CNN-ViT Hybrid	0.9843	0.98	Comparable	*p*>0.05
QGC-CNN (Proposed)	0.9463	0.945	Comparable	*p*>0.05
EfficientNetB0	0.9734	0.97	Inferior	*p* < 0.001
ConvNeXt	0.8309	0.83	Inferior	*p* < 0.001
**Stacking ensemble (proposed)**	**0.9519**	**0.95**	**–**	**–**

Bold values indicate the best-performing result for the corresponding metric.

To further analyze pairwise statistical differences, the results of McNemar's test, paired *t*-test, and Wilcoxon test are presented in Table 18.

**Table 18:** Statistical significance analysis of the proposed stacking ensemble.

Comparison	McNemar *p*-value	*t*-test *p*-value	Wilcoxon *p*-value	Conclusion
Stack vs. QGC-CNN	0.5403	0.4144	0.4142	No significant difference
Stack vs. ResNet	0.2301	0.1616	–	No significant difference
Stack vs. Xception	0.8137	0.6375	–	No significant difference
Stack vs. EfficientNet	2.99 × 10^−5^	1.47 × 10^−5^	–	Stack significantly better

In addition, bootstrap-based confidence intervals for accuracy are reported in Table 19 to assess the stability of model performance.

**Table 19:** Bootstrap 95% confidence intervals for accuracy.

Model	95% Confidence interval
Stacking ensemble	[0.9406, 0.9625]
Hybrid (QGC-CNN)	[0.9381, 0.9600]

Although the statistical results are equivalent to those obtained using the best performing baseline algorithms on the internal test data, the proposed approach exhibits consistent and robust performance. In addition, the model performs well even on unseen data, thus highlighting its ability to generalize despite changes in the underlying domain distribution. From the above analysis, the proposed algorithm is characterized by stable performance under varied testing conditions. Although the numerical gains are small, the consistency witnessed during the various statistical analyses provides evidence of robust behavior. While the analysis shows that the model performs well and robustly, this approach fails to illustrate how the model behaves when faced with difficult situations. It is for this reason that an analysis of errors is undertaken.

#### 4.9.1 Quantitative analysis of edge case misclassifications

**Figure 12:**
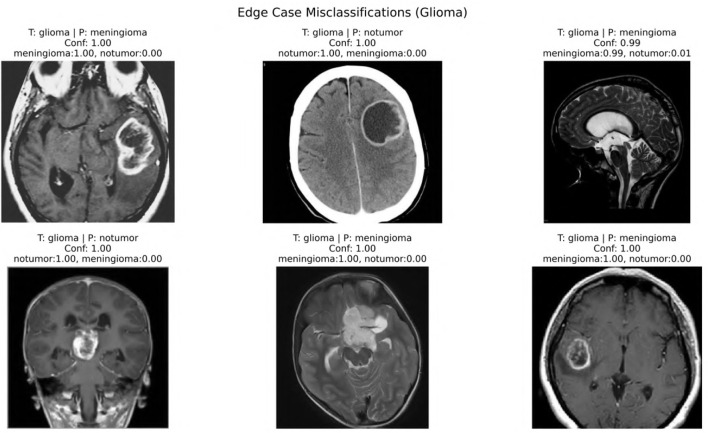
Representative edge-case misclassifications from the proposed model. Most errors occur in glioma samples, where tumors exhibit ambiguous boundaries, low contrast, or structural similarity with meningioma and normal tissue. The model often assigns high confidence to incorrect predictions, indicating that these samples lie near overlapping regions in the feature space.

Misclassification analysis was done to provide an insight into the model's weaknesses when facing difficult cases. The results of testing on 1600 data showed that only 72 samples (4.5%) were classified wrongly. It is worth mentioning that 71 out of 72 cases (98.6%) of misclassification occurred in *glioma*, which means it is the hardest class for our model. Detailed analysis shows that most cases of misclassification take place between the classes *glioma* and *meningioma* (43 instances) and *glioma* classified as *notumor* (26 samples). Very few cases of misclassification were detected in other classes, which indicates the perfection of our classifier in cases of *meningioma, notumor*, and *pituitary* classes. These findings suggest that the model's errors are not random but are concentrated in clinically challenging edge cases. In particular, glioma samples often exhibit heterogeneous structures, low contrast boundaries, and overlapping visual features with other tumor types. In some cases, low-grade gliomas may resemble normal brain tissue, leading to confusion with the *notumor* class. The misclassified samples presented in Figure 12 correspond to a selection of particularly difficult-to-classify gliomas using the model under discussion. Such samples reflect the rationale behind the occurring classification mistakes. Since all mistakes are related to the difficulty of classifying gliomas, it is safe to state that the problem lies within the nature of the data, and not within the algorithm per se. In fact, such difficulties in classification are not unusual in clinical settings where experts sometimes cannot accurately distinguish among different tumors, particularly at an early stage. These observations highlight the importance of understanding the contribution of different components in the proposed architecture. To further investigate, an ablation study is conducted in the following section.

### 4.10 Ablation studies

To systematically evaluate the contribution of key components in the proposed framework, an ablation study was conducted focusing on two major aspects: (i) the learnable query-guided attention mechanism and (ii) the stacking-based meta-learner. All experiments were performed using the same 5-fold Stratified Cross-Validation protocol, ensuring consistency across model variants.

#### 4.10.1 Baseline model comparison

As part of the initial steps in the ablation test, baselines CNNs are tested using similar experimental conditions, thus setting up a benchmark for comparison. Three popular deep convolutional neural network structures—ResNet50, Xception, and EfficientNetB0—have been assessed under similar conditions. As a result, the most successful architecture was found to be Xception, reaching the level of accuracy and macro F1-score equal to 98.57% and 0.9857 respectively, followed by ResNet50 with 98.18% accuracy. In turn, EfficientNetB0 produced inferior results, which can be explained by its inability to efficiently handle the task under consideration (82.05%). Thus, it is necessary to note that although modern CNNs are highly efficient, their performance depends heavily on the particular architecture.

#### 4.10.2 Impact of query-guided attention

In order to demonstrate the effectiveness of the attention model in isolation, a model version without attention (Hybrid-NoAttn) was implemented and scored 96.77% accuracy and 0.9675 macro F1 score. The addition of the query-guided attention mechanism (QGC-CNN) resulted in increased accuracy to 96.91% and macro F1 score to 0.9690. Although not significant, the difference between models is consistent throughout all metrics and notably helps increase recall and specificity values for the hardest to classify classes like meningioma. Thus, it can be concluded that the learnable query tokens are effective at capturing global context relationships.

#### 4.10.3 Impact of stacking-based meta-learner

In order to measure the impact of the stacking approach, a stacked model based on traditional CNN architectures (ResNet50, Xception, and EfficientNetB0) was developed, which attained an accuracy of 98.75%. Clearly, stacking of features from different CNNs results in significant gains by itself. Nevertheless, adding the proposed QGC-CNN network to the stacked model resulted in further performance increase to 98.78% (macro F1-score = 0.9879). Although the gains are relatively small numerically, introducing QGC-CNN into the process increases the feature diversity, improving performance consistency for all classes.

From the ablation results (Table 20), it is clear that each contribution plays an important role in achieving optimal performance. Query guided attention allows for better feature extraction through the addition of global context into the representation. Stacking-based meta learner plays a crucial role in improving performance through the exploitation of the benefits offered by complementary modeling. More importantly, from the ablation results, it can be observed that despite the availability of strong CNN baselines such as Xception model which are very accurate, the proposed architecture outperforms all other approaches through the integration of the query guided attention with the stacking-based meta-learner.

**Table 20:** Ablation study: evaluation of query-guided attention and stacking strategies.

Metric	No query, no attention	QGC-CNN	ResNet50	Xception	Stack (CNN)	Stack (full)
Accuracy	96.77	96.91	98.18	98.57	98.75	**98.78**
Macro F1	0.9675	0.9690	0.9817	0.9857	0.9875	**0.9879**
Recall	0.9677	0.9691	0.9817	0.9857	0.9900	**0.9879**
Specificity	0.9892	0.9897	0.9930	0.9952	0.9830	**0.9960**
MCC	0.9571	0.9588	0.9757	0.9810	0.9833	**0.9838**
Kappa	0.9569	0.9592	0.9758	0.9810	0.9833	**0.9838**
ROC-AUC	0.9985	0.9988	0.9991	**0.9995**	**0.9995**	**0.9995**
Log Loss	0.0949	0.0959	0.0700	0.0520	**0.0420**	0.0435

Bold values indicate the best-performing result for the corresponding metric.

## 5 Discussion

This section of our article consolidates the findings of our system, starting with the implications, then discusses the strengths and weaknesses of our model.

### 5.1 The efficacy of learnable query tokens

A key contribution of this work is the integration of learnable query tokens, which enable the model to jointly capture local texture information and global contextual relationships. Unlike conventional CNNs, which are limited to local receptive fields, the proposed approach leverages cross-attention to selectively focus on diagnostically relevant regions. Classical Vision Transformers (ViTs) are generally too demanding to allow for real-time applications in a clinical environment. A limited number of learnable query tokens, *k* = 16, is employed and their interaction with the feature maps to selectively focus on relevant regions for tumorous features obtained through CNNs. Rather than being particularly constrained to each class, these tokens behave as task-related latent probes, which learn to concentrate on significant tumor-related patterns that can be observed in the images. The various weights are assigned dynamically by the cross-attention operation of the model to each token based on the pathological characteristics of the image itself. This helps the network focus more selectively on different areas of the tumor while suppressing other areas that might not add any useful information for the decision-making process. This allows the model to “interrogate” the MRI slice for particular anomalies, similar to how a human radiologist inspects the image for changes in intensity values. Beyond feature extraction, it is essential to evaluate how these representations translate into reliable predictive performance, particularly in ensemble settings.

### 5.2 The reliability-accuracy paradox: prioritizing clinical safety

One of the most important observations made in our comparative analysis of ensemble paradigms is the trade-off between Bagging and Stacking with respect to performance. Although the Bagging ensemble paradigm reported the highest nominal accuracy of 0.9943, its Log Loss of 0.2819 is significantly high, indicating a deep-rooted flaw in medical diagnosis: “over-confident” misclassification. Conversely, although our proposed Stacking Ensemble paradigm reported a slightly lower accuracy of 0.9878, its Log Loss of 0.0434 is substantially better. From a medical ethics and safety point of view, the improvement in the probability calibration of our proposed Stacking Ensemble paradigm is the better-performing metric. The LightGBM meta-model was able to effectively detect and mitigate the biases of the diverse set of base models, ensuring that the final probability predictions are “trustworthy” and well-calibrated. The stacking paradigm was able to decrease the False Positive Rate while preserving a high Matthews Correlation Coefficient of 0.9838, ensuring that the paradigm has a potential for a clinical decision-support system where the “cost of a false alarm” could result in unnecessary invasive procedures.

### 5.3 Balancing performance and deployment

Although high-capacity models such as Xception and ResNet50 are capable of high diagnostic accuracy, their computational requirements (9.11 and 7.73 GFLOPs, respectively) are often prohibitive for practical use in a clinical setting. The 0.83 GFLOPs of the proposed QGC-CNN model is considered not only as a performance indicator but as a technical requirement for global healthcare equity. The 10.9-fold reduction in complexity from the Xception model ensures that the model is deployable in a resource-poor clinical setting or on a mobile diagnostic station without the need for high-end GPU clusters. With a test accuracy of 95.50% at such a low computational complexity, the QGC-CNN (stack ensemble) demonstrates that “active interrogation” with learnable query tokens can extract semantic features more efficiently than the conventional depth of convolutional neural networks. In addition to performance, interpretability plays a crucial role in clinical adoption.

### 5.4 Explainability

The clinical applicability of DL models is mostly hindered by a lack of transparency; however, our QGC-CNN design resolves this issue by incorporating task-aligned transparency. The cross-attention maps enable visual validation that the model is indeed learning actual medical anatomy and not merely artifactual noise. Specifically, for the Pituitary tumor class, the learnable query tokens exhibited high spatial precision by focusing on activations strictly at the cranial base. This focus has important clinical implications in that it corresponds directly to the anatomical site where pituitary adenomas arise. Moreover, the model's fallback plan in the “No Tumor” control group, where tokens are reset to a symmetrical anatomical scan, proves that the model is indeed “searching” for biomarkers and not merely scanning pixels. This high-fidelity alignment of semantic representations and pathological anatomy satisfies an essential criterion for clinical trustworthiness.

### 5.5 Learning distinct tumor-specific representations

To further understand the effectiveness of the learned features, the discriminative representation capability of the proposed model is analyzed. The “distinct” part of our method is attained through an integration or fusion of global and local contexts:

- Hybrid feature extraction: The EfficientNetB0 network is used for local texture aspects such as tumor density, while the Transformer Path handles long-range dependencies like the relationship with nearby structures in the brain.
- Fused representation *z*: The output is a high-dimensional feature vector represented by *z*, which concatenates the attended query embeddings with the global features of CNN.
- Discrimination power: The result of the effectiveness of these “Distinct Representations” can be demonstrated by our AUC of 1.00 on most classes on this dataset size (*N* = 5, 600), showing the distinctiveness of the learned features by achieving a near perfect class distinction.

### 5.6 Implications

The QGC-CNN framework presented here shows how learning-based queries can enable global context modeling effectively while avoiding the computationally intensive nature usually seen in transformers. As opposed to the classic CNN models that make use of only local receptive fields, the proposed method pays attention to clinically meaningful regions, just like the way radiologists do during diagnosis. Additionally, with stacking, better probabilistic calibration is made possible, which is important in medical decision-making systems because wrong decisions can be quite costly. The automation of the classification process can also assist in easing the workload of medical specialists and enhancing the efficiency of the diagnostic process. Focusing on computational efficiency, FLOPs and GPU time per batch are reported in Table 5, which can provide valuable insight into the evaluated models. Among the chosen CNN-based models, EfficientNetB0 demonstrated not only the best trade-off between speed and efficiency, but also achieved strong validation accuracy with the lowest computational cost (0.78 GFLOPs and 0.32 s/batch). On the other hand, ResNet50 achieved the highest validation accuracy across most folds; however needs a moderately higher computational demand (7.73 GFLOPs). It is evident that the Xception network is efficient in feature extraction, it incurred the highest FLOPs (9.11 GFLOPs) and a slightly longer inference time. The proposed Query-Guided Cross-Attention CNN (QGC-CNN) maintained competitive efficiency (0.83 GFLOPs) while benefiting from enhanced contextual learning, confirming that the addition of the attention pathway does not significantly increase inference cost. The proposed model is well balanced in terms of accuracy, efficiency, as well as inference speed, making it suitable candidate for real-time clinical application.

### 5.7 Analysis of the generalization gap

Although we have managed to achieve relatively high internal accuracy, it is essential to test the framework's robustness against the “Domain Shift” challenge—one of the most prevalent issues within clinical artificial intelligence. For this purpose, we performed cross-dataset validation using two separate, multi-center databases: Mendeley Dataset: The suggested ensemble demonstrated a robust level of accuracy, amounting to 93.86%, along with an excellent macro-F1 score (0.9407). This impressive result confirms the generalizability of the model when it comes to various T1-weighted contrast-enhanced sequences.

### 5.8 Clinical utility: high-specificity screening

Clinical implementation of such results is characterized by reliability of the test. For example, even in cases when the drop in the total performance leads to the reduction in overall accuracy, Recall value is still equal to 1.00 when predicting “No Tumor.” The results allow us to claim that the algorithm can serve as an efficient tool for ruling out cases without pathology. At the same time, the specificity rate of 1.00 for classes of glioma and pituitary tumors implies a rather conservative approach to diagnosis. In this case, there will be almost no risk of false positive results and related harm for patients. Therefore, the proposed tool cannot be used instead of radiologists' expertise but represents a tool for double-checking results: Consistency Test: When the results and Grad-CAM visualization are consistent, this allows us to speak about high confidence in the prediction made. While MRI-based analysis and Grad-CAM visualizations provide valuable insights, they are intended as assistive tools and do not replace definitive diagnostic procedures such as histopathological examination, which remains the clinical gold standard. Inconsistency Signal: When inconsistency between the heat map and lesions is noticeable, it signals about the presence of domain-shift problem.

### 5.9 Strengths and limitation

The primary strengths of our suggested method are: The employment of a stacked ensemble of heterogeneous models renders the system more robust and less susceptible to overfitting than a single model. The QGC-CNN architecture is a new way of effectively merging the benefits of both CNNs and transformers for medical image classification. The model obtains state-of-the-art accuracy on the Brain Tumor MRI dataset.

#### 5.9.1 Limitations

Though the model works outstandingly on the existing data, it would be pertinent to point out some limitations: The performance of deep learning models largely depends on the amount and variety of data used for training. It is the need of the hour to check the performance of the model on larger and varied datasets to determine its ability to generalize. The model needs to be checked on external data from different hospitals and machines to authenticate its potency in practical scenarios.

## 6 Conclusion

The proposed stacked ensemble approach, with the new QGC-CNN model and learnable query tokens, sets a new standard for efficient and interpretable brain tumor classification. The model's ability to reach 100% recall for Healthy Tissue (No Tumor) and perfect precision for Glioma detection on the unseen test data shows the high sensitivity needed for screening. The combination of Test-Time Augmentation (TTA) and meta-learning fusion ensures that the model is domain invariant, as domain shifts are common in multi-site MRI datasets. In the end, this approach offers a complete solution that combines high diagnostic performance with low computational complexity, closing the gap between qualitative evidence of improved separability and deep learning research and practical, deployable medical software. Future research will focus on adapting the proposed methodology to multi-modal medical imaging (t1,t2,flair) for better classification of tumors. The application of federated learning can help achieve secure model training without compromising the privacy of data shared across different hospitals. Lightweight architectures can also be explored for edge-based implementations in clinical practice involving radiologists.

## Data Availability

The data used in this study are publicly available from the Brain Tumor MRI Dataset by Nickparvar (2021) on Kaggle: https://www.kaggle.com/datasets/masoudnickparvar/brain-tumor-mri-dataset. All experiments and analyses reported in this study were conducted using this dataset.

## References

[B1] Anand V. Khajuria A. Pachauri R. K. Gupta V. (2026). Multi-class classification of brain tumors using optimized CNN and transfer learning techniques. Sci. Rep. 16:4709. doi: 10.1038/s41598-025-34806-6 41593164 PMC12868662

[B2] Arun N. Gaw N. Singh P. Chang K. Aggarwal A. Chen B. . (2021). Assessing the (un)reliability of saliency maps for localizing abnormalities in medical imaging. arXiv preprint arXiv:2008.06063. 10.1148/ryai.2021200267 PMC8637231 34870212

[B3] Barber C. B. Dobkin D. P. Huhdanpaa H. (1996). The quickhull algorithm for convex hulls. ACM Trans. Mathem. Softw. 22, 469–483. doi: 10.1145/235815.235821

[B4] Batool A. Byun Y.-C. (2025). A lightweight multi-path convolutional neural network architecture using optimal features selection for multiclass classification of brain tumor using magnetic resonance images. Results Eng. 25:104327. doi: 10.1016/j.rineng.2025.104327

[B5] Breiman L. (1996). Bagging predictors. Mach. Learn. 24, 123–140. doi: 10.1023/A:1018054314350

[B6] Carion N. Massa F. Synnaeve G. Usunier N. Kirillov A. Zagoruyko S. (2020). “End-to-end object detection with transformers,” in European Conference on Computer Vision (Springer). doi: 10.1007/978-3-030-58452-8_13

[B7] Chen J. Lu Y. Yu Q. Luo X. Adeli E. Wang Y. . (2021). Transunet: transformers make strong encoders for medical image segmentation. arXiv preprint arXiv:2102.04306.

[B8] Cheng J. Huang W. Cao S. Yang R. Yang W. Yun Z. . (2015). Enhanced performance of brain tumor classification via tumor region augmentation and partition. PLoS ONE 10:e0140381. doi: 10.1371/journal.pone.0140381 26447861 PMC4598126

[B9] Chollet F. (2017). “Xception: deep learning with depthwise separable convolutions,” in Proceedings of the IEEE Conference on Computer Vision and Pattern Recognition, 1251–1258. doi: 10.1109/CVPR.2017.195

[B10] Deng J. Dong W. Socher R. Li L. J. Li K. Fei-Fei L. (2009). “Imagenet: a large-scale hierarchical image database,” in 2009 IEEE Conference on Computer Vision and Pattern Recognition (IEEE), 248–255. doi: 10.1109/CVPR.2009.5206848

[B11] Dosovitskiy A. (2020). An image is worth 16x16 words: transformers for image recognition at scale. arXiv preprint arXiv:2010.11929.

[B12] Dosovitskiy A. Beyer L. Kolesnikov A. Weissenborn D. Zhai X. Unterthiner T. . (2021). An image is worth 16x16 words: transformers for image recognition at scale. arXiv preprint arXiv:2010.11929.

[B13] Efron B. (1992). “Bootstrap methods: another look at the jackknife,” in Breakthroughs in Statistics: Methodology and Distribution (Springer), 569–593. doi: 10.1007/978-1-4612-4380-9_41

[B14] Freund Y. Schapire R. E. (1997). A decision-theoretic generalization of on-line learning and an application to boosting. J. Comput. Syst. Sci. 55, 119–139. doi: 10.1006/jcss.1997.1504

[B15] Geisser S. (1975). The predictive sample reuse method with applications. J. Am. Stat. Assoc. 70, 320–328. doi: 10.1080/01621459.1975.10479865

[B16] Goldbrunner R. Minniti G. Preusser M. Jenkinson M. D. Sallabanda K. Houdart E. . (2016). Eano guidelines for the diagnosis and treatment of meningiomas. Lancet Oncol. 17, e383–e391. doi: 10.1016/S1470-2045(16)30321-7 27599143

[B17] Golkarieh A. Boroujeni S. R. Kiashemshaki K. Deldadehasl M. Aghayarzadeh H. Ramezani A. (2025). Breakthroughs in brain tumor detection: leveraging deep learning and transfer learning for MRI-based classification. Comput. Decis. Mak. 2, 708–722. doi: 10.59543/comdem.v2i.14243

[B18] Gómez-Guzmán M. A. Jiménez-Beristain L. García-Guerrero E. E. Aguirre-Castro O. A. Esqueda-Elizondo J. J. Ramos-Acosta E. R. . (2025). Enhanced multi-class brain tumor classification in MRI using pre-trained cnns and transformer architectures. Technologies 13:379. doi: 10.3390/technologies13090379

[B19] Hamada A. (2020). Br35h: Brain tumor detection 2020. Available online at: https://www.kaggle.com/datasets/ahmedhamada0/brain-tumor-detection (Accessed February 15, 2026).

[B20] Hastie T. Tibshirani R. Friedman J. (2009). The Elements of Statistical Learning: Data Mining, Inference, and Prediction. New York: Springer Science &Business Media. doi: 10.1007/978-0-387-84858-7

[B21] He K. Zhang X. Ren S. Sun J. (2016). “Deep residual learning for image recognition,” in Proceedings of the IEEE Conference on Computer Vision and Pattern Recognition, 770–778. doi: 10.1109/CVPR.2016.90

[B22] Heythem B. Djerioui M. Beghriche T. Zerguine A. Beghdadi A. (2024). Customized cnn for multi-class classification of brain tumor based on MRI images. Arabian J. Sci. Eng. 49, 16903–16918. doi: 10.1007/s13369-024-09284-z

[B23] Hira M. I. K. Hossain M. S. Bithee M. M. A. Sara U. S. Hasan M. M. Towsif A. A. . (2026). Brain tumor MRI dataset (glioma, meningioma, pituitary, no tumor). Mendeley Data, 1, 2025.

[B24] Hosny K. M. Mohammed M. A. Salama R. A. Elshewey A. M. (2025). Explainable ensemble deep learning-based model for brain tumor detection and classification. Neural Comput. Applic. 37, 1289–1306. doi: 10.1007/s00521-024-10401-0

[B25] Hussain M. I. Chowdhury S. H. Hossain M. M. Mamun M. (2026). Neuroblend-3: Hybrid deep and machine learning framework with explainable AI for multi-class brain tumor detection using MRI scans. Medinformatics 3, 56–66. doi: 10.47852/bonviewMEDIN52026540

[B26] Hussain S. S. Wani N. A. Kaur J. Ahmad N. Ahmad S. (2025). Next-generation automation in neuro-oncology: advanced neural networks for MRI-based brain tumor segmentation and classification. IEEE Access 13, 41141–41158. doi: 10.1109/ACCESS.2025.3547796

[B27] Joshi K. P. Gowda V. B. Divakarachari P. B. Parameshwarappa P. S. Patra R. K. (2025). VSA-GCNN: attention guided graph neural networks for brain tumor segmentation and classification. Big Data Cogn. Comput. 9:29. doi: 10.3390/bdcc9020029

[B28] Ke G. Meng Q. Finley T. . (2017). “Lightgbm: a highly efficient gradient boosting decision tree,” in Advances in Neural Information Processing Systems, 3146–3154.

[B29] Kickingereder P. Burth S. Wick A. Götz M. Eidel O. Schlemmer H.-P. . (2016). Radiomic profiling of glioblastoma: identifying an imaging predictor of patient survival with improved performance over established clinical and radiologic risk models. Radiology 280, 880–889. doi: 10.1148/radiol.2016160845 27326665

[B30] Kohavi R. (1995). “A study of cross-validation and bootstrap for accuracy estimation and model selection,” in International Joint Conference on Artificial Intelligence (IJCAI), 1137–1145.

[B31] Lai Y. Cao A. Gao Y. Shang J. Li Z. (2025). Advancing efficient brain tumor multi-class classification: New insights from the vision mamba model in transfer learning. Int. J. Imaging Syst. Technol. 35:e70177. doi: 10.1002/ima.70177

[B32] Law M. Yang S. Wang H. Babb J. S. Johnson G. Cha S. . (2003). Glioma grading: sensitivity, specificity, and predictive values of perfusion MR imaging and proton MR spectroscopic imaging compared with conventional mr imaging. Am. J. Neuroradiol. 24, 1989–1998. 14625221 PMC8148904

[B33] Lin M. Chen Q. Yan S. (2013). Network in network. arXiv preprint arXiv:1312.4400.

[B34] Liu Z. Lin Y. Cao Y. Hu H. Wei Y. Zhang Z. . (2021). “Swin transformer: hierarchical vision transformer using shifted windows,” in Proceedings of the IEEE/CVF International Conference on Computer Vision (ICCV), 10012–10022. doi: 10.1109/ICCV48922.2021.00986

[B35] Louis D. N. Perry A. Reifenberger G. Von Deimling A. Figarella-Branger D. Cavenee W. K. . (2016). The 2016 world health organization classification of tumors of the central nervous system: a summary. Acta Neuropathol. 131, 803–820. doi: 10.1007/s00401-016-1545-1 27157931

[B36] Louis D. N. Perry A. Wesseling P. Brat D. J. Cree I. A. Figarella-Branger D. . (2021). The 2021 who classification of tumors of the central nervous system: a summary. Neuro-oncology 23, 1231–1251. doi: 10.1093/neuonc/noab106 34185076 PMC8328013

[B37] Lundberg S. M. Erion G. Chen H. DeGrave A. Prutkin J. M. Nair B. . (2020). From local explanations to global understanding with explainable ai for trees. Nat. Mach. Intell. 2, 56–67. doi: 10.1038/s42256-019-0138-9 32607472 PMC7326367

[B38] Maloji S. (2024). Optimised resnet50 for multi-class classification of brain tumors. Scal. Comput. 25, 1667–1680. doi: 10.12694/scpe.v25i3.2707

[B39] Matthews B. W. (1975). Comparison of the predicted and observed secondary structure of t4 phage lysozyme. Biochim. Biophys. Acta 405, 442–451. doi: 10.1016/0005-2795(75)90109-9 1180967

[B40] McNemar Q. (1947). Note on the sampling error of the difference between correlated proportions. Psychometrika 12, 153–157. doi: 10.1007/BF02295996 20254758

[B41] Molitch M. E. (2017). Diagnosis and treatment of pituitary adenomas: a review. JAMA 317, 516–524. doi: 10.1001/jama.2016.19699 28170483

[B42] Muksimova S. Umirzakova S. Mardieva S. Iskhakova N. Sultanov M. Cho Y. I. (2025). A lightweight attention-driven yolov5m model for improved brain tumor detection. Comput. Biol. Med. 188:109893. doi: 10.1016/j.compbiomed.2025.109893 39987698

[B43] Nancy A. M. Maheswari R. (2025). Brain tumor segmentation and classification using transfer learning based cnn model with model agnostic concept interpretation. Multimed. Tools Appl. 84, 2509–2538. doi: 10.1007/s11042-024-20353-1

[B44] Nassar S. E. Yasser I. Amer H. M. Mohamed M. A. (2024). A robust MRI-based brain tumor classification via a hybrid deep learning technique. J. Supercomput. 80, 2403–2427. doi: 10.1007/s11227-023-05549-w

[B45] Nickparvar M. (2021). Brain tumor MRI dataset. Kaggle. Available at: https://www.kaggle.com/datasets/masoudnickparvar/brain-tumor-MRI-dataset (Accessed February 22, 2026).

[B46] Prabhas K. S. Basem A. Lakshmi L. Talha A. Mohammed S. H. Khan M. I. . (2025). A deep learning framework for brain tumor detection using cnns and transfer learning on MRI scans. Syst. Soft Comput. 7:200389. doi: 10.1016/j.sasc.2025.200389

[B47] Prechelt L. (1998). “Early stopping—but when?,” in Neural Networks: Tricks of the Trade (Berlin, Heidelberg: Springer Berlin Heidelberg), 55–69. doi: 10.1007/3-540-49430-8_3

[B48] Rousseeuw P. J. (1987). Silhouettes: a graphical aid to the interpretation and validation of cluster analysis. J. Comput. Appl. Math. 20, 53–65. doi: 10.1016/0377-0427(87)90125-7

[B49] Sagi O. Rokach L. (2018). Ensemble learning: a survey. Data Min. Knowl. Disc. 8:e1249. doi: 10.1002/widm.1249

[B50] Sartaj B. (2020). Brain Tumor Classification (MRI). Kaggle.

[B51] Schapire R. E. (2013). “Explaining adaboost,” in Empirical inference: Festschrift in honor of Vladimir Vapnik, 37–52. doi: 10.1007/978-3-642-41136-6_5

[B52] Selvaraju R. R. Cogswell M. Das A. Vedantam R. Parikh D. Batra D. (2017). “Grad-CAM: visual explanations from deep networks via gradient-based localization,” in Proceedings of the IEEE International Conference on Computer Vision (ICCV), 618–626. doi: 10.1109/ICCV.2017.74

[B53] Stone M. (1974). Cross-validatory choice and assessment of statistical predictions. J. R. Stat. Soc. 36, 111–133. doi: 10.1111/j.2517-6161.1974.tb00994.x

[B54] Student (1908). The probable error of a mean. Biometrika 1908, 1–25. doi: 10.2307/2331554

[B55] Tan M. Le Q. V. (2019). “Efficientnet: rethinking model scaling for convolutional neural networks,” in International Conference on Machine Learning (PMLR), 6105–6114.

[B56] Tariq A. Iqbal M. M. Javed Iqbal M. Ahmad I. (2025). Transforming brain tumor detection empowering multi-class classification with vision transformers and efficientnetv2. IEEE Access 13, 63857–63876. doi: 10.1109/ACCESS.2025.3555638

[B57] Tjoa E. F. Guan C. (2020). A survey on explainable artificial intelligence (xAI): toward medical xai. IEEE Trans. Neural Netw. Learn. Syst. 32, 4793–4813. doi: 10.1109/TNNLS.2020.3027314 33079674

[B58] van der Maaten L. Hinton G. (2008). Visualizing data using t-SNE. J. Mach. Learn. Res. 9, 2579–2605.

[B59] Vaswani A. Shazeer N. Parmar N. Uszkoreit J. Jones L. Gomez A. N. . (2017). “Attention is all you need,” in Advances in Neural Information Processing Systems, 5998–6008.

[B60] Wang G. Li W. Dogra P. N. Dasgupta A. Kulkarni R. Davidson A. J. . (2019). Aleatoric uncertainty estimation with test-time augmentation for medical image segmentation with convolutional neural networks. Neurocomputing 338, 34–45. doi: 10.1016/j.neucom.2019.01.103 31595105 PMC6783308

[B61] Wilcoxon F. (1992). “Individual comparisons by ranking methods,” in Breakthroughs in Statistics: Methodology and Distribution (Springer), 196–202. doi: 10.1007/978-1-4612-4380-9_16

[B62] Wolpert D. H. (1992). Stacked generalization. Neural Netw. 5, 241–259. doi: 10.1016/S0893-6080(05)80023-1

[B63] Yang Y. Lv H. Chen N. (2023). A survey on ensemble learning under the era of deep learning. Artif. Intell. Rev. 56, 5545–5589. doi: 10.1007/s10462-022-10283-5

[B64] Zarenia E. Far A. A. Rezaee K. (2025). Automated multi-class MRI brain tumor classification and segmentation using deformable attention and saliency mapping. Sci. Rep. 15:8114. doi: 10.1038/s41598-025-92776-1 40057634 PMC11890586

